# Rapid Identification of Bacillus anthracis *In Silico* and On-Site Using Novel Single-Nucleotide Polymorphisms

**DOI:** 10.1128/spectrum.02285-21

**Published:** 2022-05-16

**Authors:** Yufei Lyu, Dongshu Wang, Lu Yuan, Erling Feng, Li Zhu, Chao Pan, Yan Guo, Xiankai Liu, Hengliang Wang

**Affiliations:** a State Key Laboratory of Pathogens and Biosecurity, Beijing Institute of Biotechnology, Beijing, China; b College of Food Science and Technology, Shanghai Ocean University, Shanghai, China; Labcorp

**Keywords:** *Bacillus anthracis*, *Bacillus cereus* group, SNP, detection, Cas12a

## Abstract

Bacillus anthracis is a spore-forming bacterium that causes life-threatening infections in animals and humans and has been used as a bioterror agent. Rapid and reliable detection and identification of B. anthracis are of primary interest for both medical and biological threat-surveillance purposes. Few chromosomal sequences provide enough polymorphisms to clearly distinguish B. anthracis from closely related species. We analyzed 18 loci of the chromosome of B. anthracis and discovered eight novel single-nucleotide polymorphism (SNP) sites that can be used for the specific identification of B. anthracis. Using these SNP sites, we developed software—named AGILE V1.1 (*a*nthracis *g*enome-based *i*dentification with high-fide*l*ity *E*-probe)—for easy, user-friendly identification of B. anthracis from whole-genome sequences. We also developed a recombinase polymerase amplification-Cas12a-based method that uses nucleic acid extracts for the specific, rapid, in-the-field identification of B. anthracis based on these SNPs. Via this method and B. anthracis-specific CRISPR RNAs for the target CR5_2, CR5_1, and Ba813 SNPs, we clearly detected 5 aM genomic DNA. This study provides two simple and reliable methods suitable for use in local hospitals and public health programs for the detection of B. anthracis.

**IMPORTANCE** Bacillus anthracis is the etiologic agent of anthrax, a fatal disease and a potential biothreat. A specific, accurate, and rapid method is urgently required for the identification of B. anthracis. We demonstrate the potential of using eight novel SNPs for the rapid and accurate detection of B. anthracis via *in silico* and laboratory-based testing methods. Our findings have important implications for public health responses to disease outbreaks and bioterrorism threats.

## INTRODUCTION

The soil bacteria Bacillus cereus, Bacillus thuringiensis, and Bacillus anthracis, members of the B. cereus group of strains, have similar spore formation and genetic characteristics to the extent that they have been considered members of the same species (1). B. cereus is known mainly as a food poisoning bacterium that causes diarrhea and vomiting but may also cause more severe infections, and B. thuringiensis is an insect pathogen. B. anthracis is the etiological agent of anthrax, a disease with high lethality in many animal species, including humans. B. anthracis was considered for use as a potential biowarfare agent during the age of the Cold War and, in later years, as a bioterror agent. Therefore, it is important that we are able to unambiguously distinguish B. anthracis isolates from other members of the B. cereus group at the genetic level.

Wild-type B. anthracis normally harbors two virulence plasmids, pXO1 and pXO2. Some genes on the plasmids have been used as targets to detect B. anthracis in PCR assays (2, 3). However, data gathered in the past decade have shown that these plasmids may be lost from some B. anthracis strains, and some B. cereus strains can acquire pXO1/pXO2-like plasmids (4, 5). This indicates that we cannot reliably identify B. anthracis using only the plasmid sequences. The mobile nature of the plasmids in B. cereus group strains emphasizes the importance of applying chromosomal markers to differentiate B. anthracis from the rest of the B. cereus group. Compared with targets on the two virulent plasmids, targets on chromosomes are more stable and more appropriate for identifying B. anthracis.

Previously, a few targets on the chromosome of B. anthracis have been considered PCR markers. Target genes have included Ba813 (6), *gyrA* (7), *gyrB* (8), *rpoB* (9), SG850 (10), *ptsI* (11), *purA* (12), and *plcR* (13). The multilocus sequencing typing (MLST) loci (*glpF*, *gmk*, *ilvD*, *pta*, *pur*, *pyc*, and *tpi*) used for molecular typing of the B. cereus group also possess the potential to be identifying markers for B. anthracis (14). The CRISPR system is a widespread immune system in bacteria that holds a record of bacterial evolution (15, 16), and CRISPR loci also have the potential to distinguish B. anthracis. We found three CRISPR sites (CRISPR 2, 3, and 5) in B. anthracis strain Ames (GI: 30260195) using the CRISPR finder program (https://crispr.i2bc.paris-saclay.fr/Server/). The recent availability of numerous whole-genome sequences of B. cereus group strains provided a basis for the reassessment of these loci with regard to whether they can be used as markers to identify B. anthracis. It is worth analyzing these loci and exploring the use of single-nucleotide polymorphisms (SNPs) specific to B. anthracis because accurate and stable SNPs can be used to develop detection methods for the strain.

B. anthracis represents a significant public health and veterinary threat and can be used in bioterror attacks, and therefore, rapid in-the-field detection of this pathogen is important. The synthetic biologist Collins first introduced the use of CRISPR/Cas technology for nucleic acid molecular diagnosis (17), and CRISPR/Cas shows excellent sequence recognition capacity. DNA endonuclease-targeted CRISPR trans-reporter (DETECTR) (18) and one-*ho*ur *l*ow-cost *m*ultipurpose highly *e*fficient *s*ystem (HOLMES) (19) can be used to detect DNA sequences from human papillomaviruses in patient samples and both the pseudorabies virus and Japanese encephalitis virus with attomolar sensitivity and high specificity via CRISPR-Cas12a, respectively. Recombinase polymerase amplification (RPA) does not require template denaturation; can be run at a low, constant temperature; and has been successfully integrated into different detection platforms (20). Integration of the CRISPR/Cas system with RPA has huge application prospects in the field of on-site testing that does not require precision instruments.

We aimed to develop reliable methods for the identification of B. anthracis in our study. First, computer-based comparative analysis of target loci was conducted aiming to identify SNP markers specific for B. anthracis. We uncovered eight novel SNPs from these loci and developed publicly available software for the rapid identification of B. anthracis based on these SNPs. Then, we used these SNPs to develop a practical and rapid on-site B. anthracis detection method that employs the Cas12a detection system combined with RPA.

## RESULTS

### Initial screening of B. anthracis genome database.

**FIG 1:**
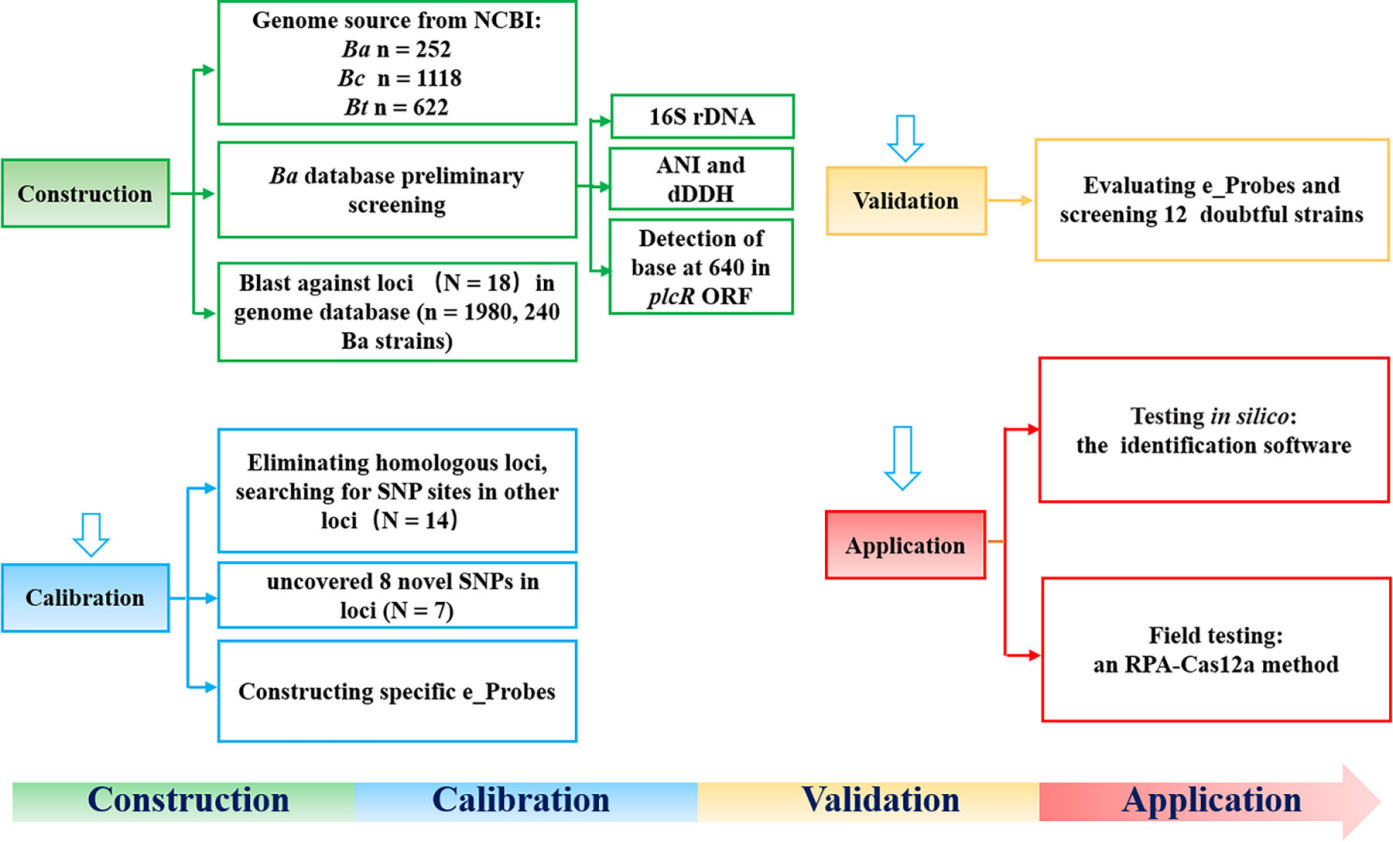
Development of Bacillus anthracis identification methods *in silico* and for field testing. *Ba*, B. anthracis; *Bc*, B. cereus; *Bt*, B. thuringiensis.

**FIG 2:**
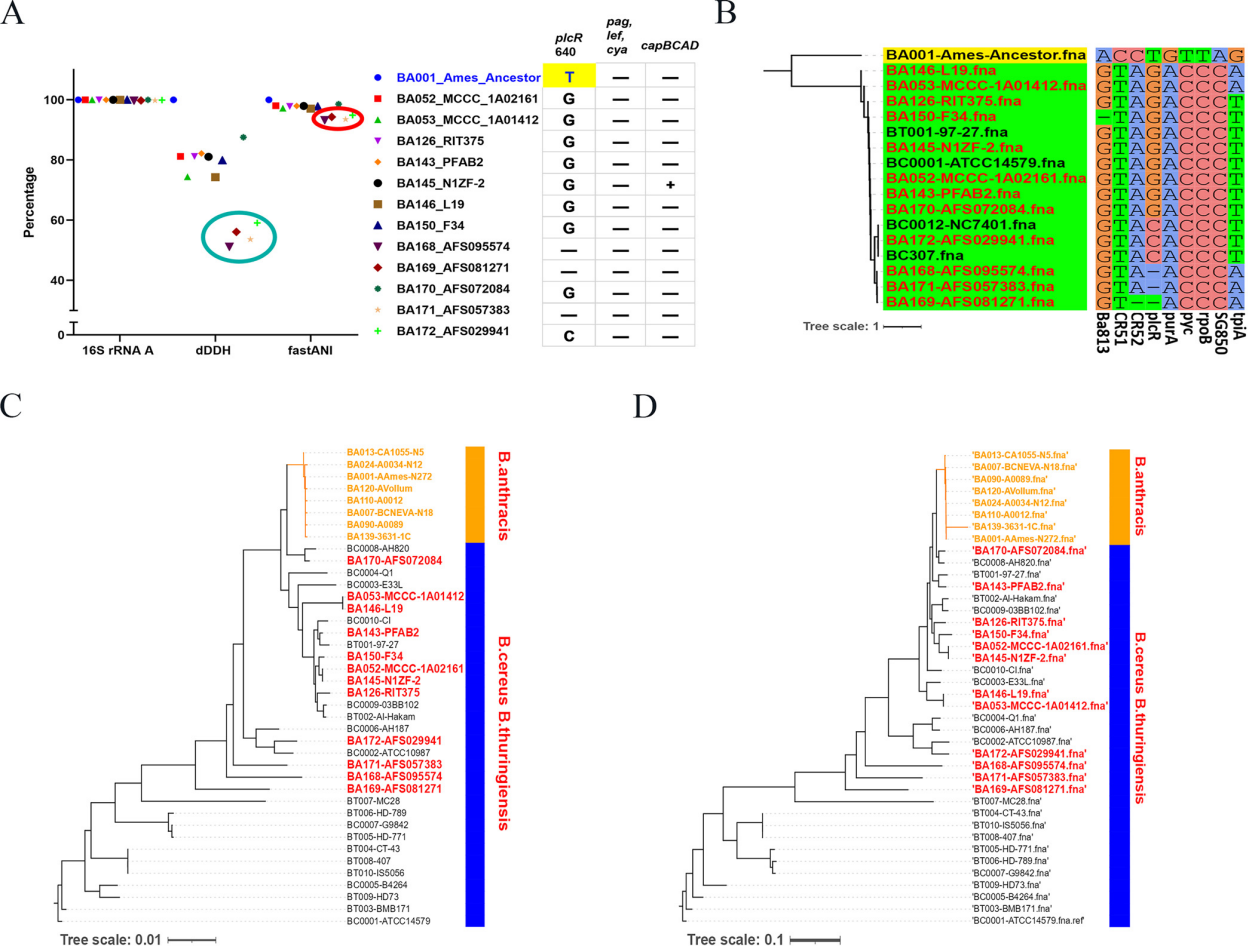
The 12 strains assigned as “B. anthracis” in the NCBI genome database were misidentified using discovered novel single-nucleotide polymorphisms (SNPs) specific to B. anthracis and software developed using these SNPs. (A) After 16S rRNA gene sequence analysis, calculation of average nucleotide identity and digital DNA-DNA hybridization values, and detection of the base at position 640 in the open reading frame of the *plcR* gene, 12 doubtful B. anthracis strains were eliminated from the strain set. –, no base could be detected at this site. (B) Display of *a*nthracis *g*enome-based *i*dentification with high-fide*l*ity *E*-probe (AGILE) V1.1 software analysis for identification of B. anthracis. The 12 doubtful B. anthracis strains, BC307, and NC7401 were revealed to be B. cereus by AGILE V1.1 scanning. The corresponding table of the assembly identifiers (IDs) and naming information for B. cereus, B. thuringiensis, and B. anthracis in this study is shown as Table S7 in the supplemental material. (C and D) The 12 doubtful B. anthracis strains were proven to be B. cereus via multilocus sequencing typing (C) and whole-genome SNP (D) analysis.

As of 10 August 2020, there were 1,992 genomes of common B. cereus sensu lato group members in the NCBI database, including 252 whole-genome sequences assigned as B. anthracis, 1,118 as B. cereus, and 622 as B. thuringiensis (see Table S6 in the supplemental material), and they were all adopted as the genomic databases for screening for specific SNPs. Among them, the genomic sequences of the other 251 B. anthracis strains were compared with the sequence of the reference strain B. anthracis Ames Ancestor (accession no. NC_007530) (Fig. 1). These strains have different genome sequencing completion statuses; some have been completely assembled, some still consist of many contigs, and some even have >1,500 contigs. To facilitate identification, the strains were renumbered (Table S7), and the 16S rRNA gene sequence, average nucleotide identity (ANI) and digital DNA-DNA hybridization (dDDH) values, and a “T” as base 640 of the *plcR* open reading frame (ORF) (13) were used in the preliminary screening of the B. anthracis strains. The results showed that all 16S rRNA gene similarities between the 251 strains in the B. anthracis database and B. anthracis Ames Ancestor were >99%. Four strains (BA168_AFS095574, BA169_AFS081271, BA171_AFS057383, and BA172_AFS029941) were removed from our database because the ANI and dDDH values were below 96% and 70%, respectively (Fig. 2A). In 12 strains, base 640 in the *plcR* ORF was not “T,” and three of the strains had a base deletion at this position. The genomes of 11 of the 12 strains possessed neither pXO1 nor pXO2 plasmids, nor any virulence factors of B. anthracis (including *pagA*, *lef*, *cya*, and *capBCAD*), while one strain (BA145_N1ZF-2; identifier [ID], GCA_001883885.1) carried *capBCAD* genes on a plasmid. Thus, we temporarily eliminated these 12 doubtful B. anthracis strains from the strain set, leaving 240 B. anthracis strains (Fig. 1 and 2A).

### Evaluation and validation of B. anthracis-specific SNPs.

To find SNPs that can be used to identify B. anthracis, we evaluated 18 loci on the chromosome, including seven MLST loci, three CRISPR loci, and eight loci previously suggested in the literature (Fig. 1; Table S1). After the 12 doubtful B. anthracis strains were eliminated, the nucleotide sequences of these loci in the 240 putative B. anthracis strains (*n *= 1,980) were analyzed by local BLAST searches using blast-2.7.1+. After alignment, the four loci (*gmk*, *gyrB*, *pta*, and *pur*) were found to be completely homologous among some B. anthracis, B. cereus, and B. thuringiensis strains and were eliminated from the locus set (*n* = 14, Fig. S1). Further analysis found six loci (crispR2, crispR3, *glpF*, *gyrA*, *ilvD*, and *ptsI*) that had no specific SNP in B. anthracis that could be distinguished from those of the B. cereus*-*B. thuringiensis strain and thus were also eliminated (*n* = 8, Fig. S2). Specific SNP sites that can be used to distinguish B. anthracis from B. cereus*-*B. thuringiensis strains were identified in eight loci (one in each of Ba813, *plcR*, *purA*, *pyc*, *rpoB*, SG850, and *tpiA* and two in CRISPR5) (Table 1; Fig. S3). Because the SNP in *plcR* was the same as that previously reported (13), it was used only to prove the accuracy of the eight novel SNP sites (*n* = 7) identified in this study. Nine specific probes based on the nine SNPs, namely, eProbes_Ba for B. anthracis and eProbes_Bct for B. cereus and B. thuringiensis (see Materials and Methods), were constructed for the convenience of description and utilization (Table 1 and Table S2).

**TABLE 1:** Information on SNPs and eProbes used in this study^*a*^

Locus	Base change	SNP position	eProbe start	eProbe end	Nucleotide sequence of eProbe
Ba813	A-G	4564265(−)	4564240	4564289	CCATTGCTAATGTATGCGAATTTC**A**ATTTGCCAAATGACAATTTAGGTTT (SEQ ID no. 1)
crispR5_1 (CR5_1)	C-T	5015039(−)	5015014	5015063	AGGTGCTGCAAAGGCAACGAATGC**C**TCTGGTAAAAAAGCTGATGACAAAT (SEQ ID no. 2)
crispR5_2 (CR5_2)	C-A	5014970(−)	5014945	5014994	AACGAGCACGACACCAGAAAGTAA**C**ACAGAAGATAAGTCTCAAAAAGAAG (SEQ ID no. 3)
*pyc*	T-C	3810008(−)	3810003	3810052	AATGCCAGGCGGACAGTACAGTAATTTACAACAACAAGCGAAAG**T**GGTTG (SEQ ID no. 4)
*rpoB*	T-C	109410(+)	109399	109448	GTGGTAGAAGG**T**GATGTTGAGCTGCAATCTATTAAGATTTATGCTCCTGA (SEQ ID no. 5)
SG850	A-C	1491472(+)	1491458	1491507	AGCGGGTGTAGGAA**A**CGGGAAAACAATTGTATATCTTCTATATGCAATTT (SEQ ID no. 6)
*tpiA*	G-T/A	4861968(−)	4861943	4861992	TCCAGCTCTATTCTTAGAGCGCCT**G**GTAGCAGCGACTGAAGGAACTGATT (SEQ ID no. 7)
*purA*	G-A	5207983(−)	5207958	5208007	ATATGTATGTGATACGTCTGTTGT**G**TTAAATGATGCATTAGATAACAATC (SEQ ID no. 8)
*plcR*	T-G/C	5081303(−)	5081278	5081327	TTATACTTGGACAATCAATACGAA**T**AAGCGCTTTGTCATGCAAATAAAGC (SEQ ID no. 9)

a Bold and underlined letters indicate SNP sites. SNP position is based on the chromosome of B. anthracis Ames Ancestor strain (NC_007530). SEQ ID, sequence identifier.

We retrospectively analyzed the genomes of 1,118 B. cereus and 622 B. thuringiensis strains to determine the specificity and effectiveness of the two electronic probe (eProbe) sets (Fig. 1); none of the SNPs included in eProbes_Ba were detected *in silico* in these B. cereus and B. thuringiensis strains. We next analyzed the genomes of 4,707 *Bacillus* strains from 87 species in local and NCBI genome databases, not including B. anthracis, as a test set (Table S6) to assess the applicability of the two eProbe sets. None of the nine SNPs in eProbes_Ba were detected in these strains (data not shown). The above results suggested that the nine SNPs in eProbes_Ba can be used to distinguish B. anthracis from its genetic near-neighbors B. cereus and B. thuringiensis.

### Development of identification software based on eProbes.

Encouraged by the specificity and effectiveness of the two eProbe sets, we compiled software for the rapid identification of B. anthracis from genome sequence data using eProbes_Ba and eProbes_Bct. The software was named *a*nthracis *g*enome-based *i*dentification with high-fide*l*ity *E*-probe (AGILE V1.1). As long as one of the eProbes_Ba is matched exactly by AGILE, the strain is identified as B. anthracis, and the more matched probes there are, the more reliable the positive result. This software can be downloaded by anyone for free from https://github.com/844844/AGILE for Windows and https://github.com/844844/Identify_B.anthracis for Python users. The identification of strains can be completed with one click by inputting sequence data (a complete sequence assembly) on a Windows-based PC. AGILE is a convenient, user-friendly tool for microbiologists, clinicians, and industry professionals.

After successful completion of the software development, the 12 doubtful B. anthracis strains (genome sequences available at https://www.github.com/844844/AGILE) were confirmed to be non-B. anthracis strains using AGILE V1.1 scanning (Fig. S4). This result is the same as that of our preliminary screening with 16S RNA, ANI, and *plcR* ORF described previously. The genomes of these 12 strains did not harbor any of the SNPs in eProbes_Ba but harbored all of those in eProbes_Bct (Fig. 2B). The SNP sites in Ba813, CR52, and *plcR* were also not harbored in several strains. The 12 strains also clustered with B. cereus and B. thuringiensis after minimum evolution method phylogenetic analysis.

To verify these results, we combined these 12 strains with 10 strains each of B. anthracis, B. cereus, and B. thuringiensis as a validation set for whole-genome phylogenetic analysis. In the resulting unweighted pair group method with arithmetic means (UPGMA) tree, the 10 B. anthracis strains clustered into one branch, and the 12 doubtful strains clustered with the 10 B. cereus strains and 10 B. thuringiensis strains in MLST analysis (seven loci: https://pubmlst.org/bigsdb?db=pubmlst_bcereus_seqdef&page=downloadAlleles&tree=1) (Fig. 2C) and whole-genome SNP (wgSNP) phylogenetic analysis (Fig. 2D). Thus, these 12 strains were not B. anthracis but may be B. cereus or B. thuringiensis. Hence, using AGILE V1.1, we further confirmed that the 12 strains assigned as “B. anthracis” in the NCBI genome database had been misidentified, even though their genome sequences were complete.

### Application of B. anthracis rapid identification software.

**FIG 3:**
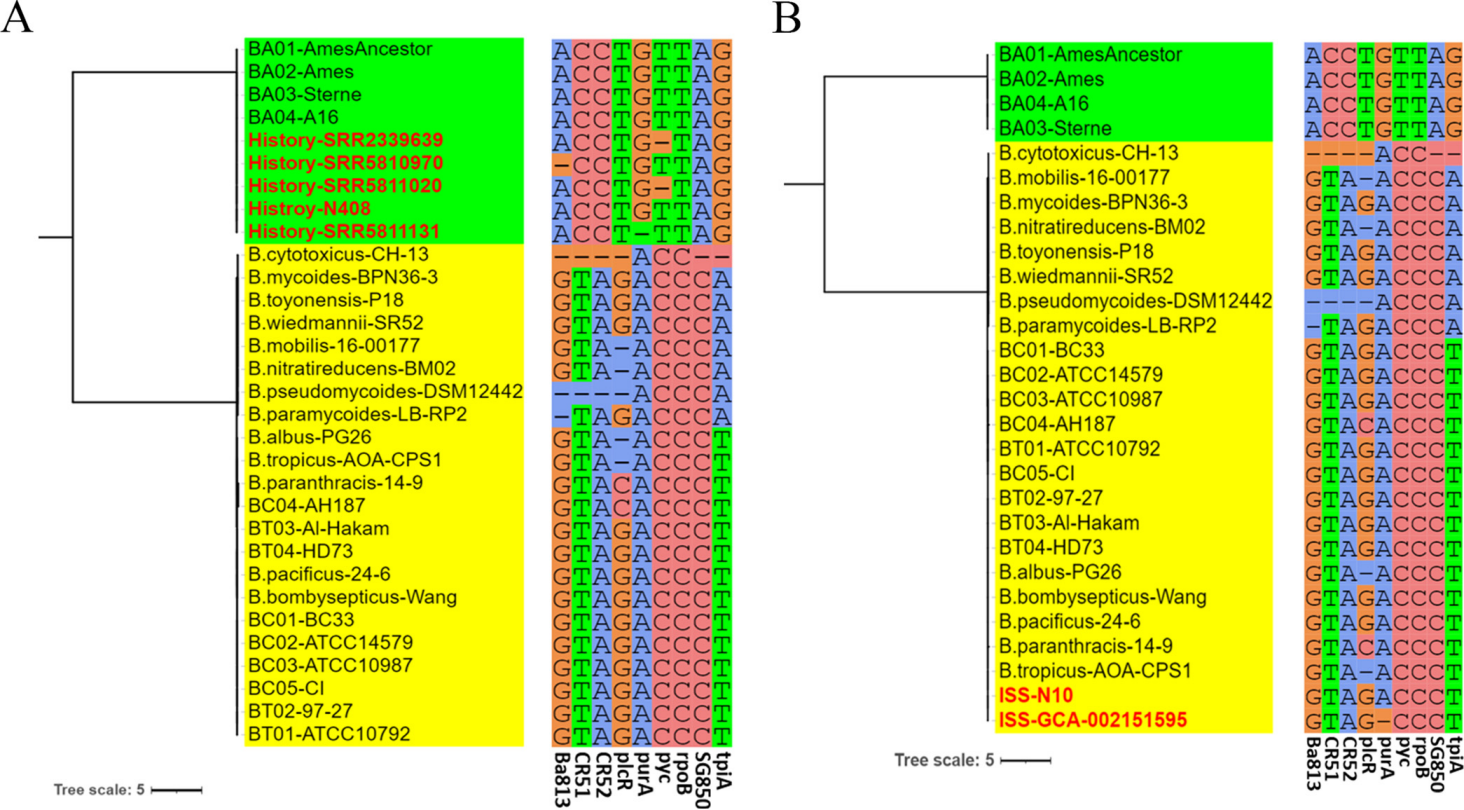
Strains with genomic information collected from the literature were used as a test set to evaluate the AGILE software. We used these genomes and representative publicly available genomes of B. cereus sensu lato strains (from the NCBI database) to generate a maximum-likelihood tree. Tree visualizations were performed using iTOL. (A) Historical strains previously assigned as B. anthracis (*n* = 412) were placed in the same cluster as B. anthracis, consistent with AGILE analysis. “History-N408” means that all nine SNP sites in our set were identical in 408 of the strains. (B) Eleven isolates from the International Space Station were identified as B. cereus and/or B. thuringiensis, not B. anthracis, by AGILE analysis. These 11 strains clustered with B. cereus and B. thuringiensis according to SNP type. “ISS-N10” means that all nine SNP sites in our set were identical in 10 of the strains. The corresponding table of the assembly identifiers and naming information for the representative strains in B. cereus sensu lato in this study is shown as Table S8 in the supplemental material.

We investigated the nucleic acid information on a historical collection of 412 B. anthracis strains in previously published literature (21); these strains were used as a test set to further evaluate our software. All the strains were identified as B. anthracis using AGILE V1.1 (Fig. 3A). The 412 strains (the remaining four strains and History-N408) are marked in red in Fig. 3A. All nine SNP sites in our set were identical in 408 of the strains (History-N408). The eProbes of Ba813, *purA* and *pyc*, were not detected in the remaining four strains, which may be related to the quality of the sequencing.

In addition to B. cereus, B. thuringiensis, and B. anthracis, the B. cereus sensu lato group includes less-common members such as Bacillus mycoides, Bacillus pseudomycoides, Bacillus weihenstephanensis, Bacillus cytotoxicus, and Bacillus toyonensis (22, 23). We used the 412 genomes mentioned above and publicly available genomes of representative strains (Table S8) of B. cereus sensu lato to generate a maximum-likelihood tree. The 412 strains were placed in the same cluster as B. anthracis, which is consistent with the AGILE analysis. Non-B. anthracis strains of B. cereus sensu lato formed a separate cluster in the maximum-likelihood tree, in agreement with the results of AGILE.

Furthermore, we collected genomic information on 11 *Bacillus* strains isolated on the International Space Station (ISS; from the experimental modules of three different countries) (24). Previous research (24) indicated that the 11 ISS isolates could be B. anthracis based on ANI and dDDH values and *gyrB* sequences, but these strains were confirmed as not being B. anthracis when later phenotypic trait and whole-genome analyses were used. After the genome sequences were entered into the AGILE software, these 11 ISS strains were identified as B. cereus or B. thuringiensis (Fig. 3B). The 11 ISS strains are marked in red in Fig. 3B. All nine SNP sites in our set were identical in 10 of the strains; the eProbe sequence in *purA* was absent from the genomic information for one strain (ISS-GCA-002151595). The 11 ISS strains clustered together with B. cereus and B. thuringiensis, among others, in a maximum-likelihood tree. Our findings indicate that AGILE is an accurate and powerful method for identifying B. anthracis.

### Detection of B. anthracis based on novel SNP sites by RPA combined with Cas12a.

**FIG 4:**
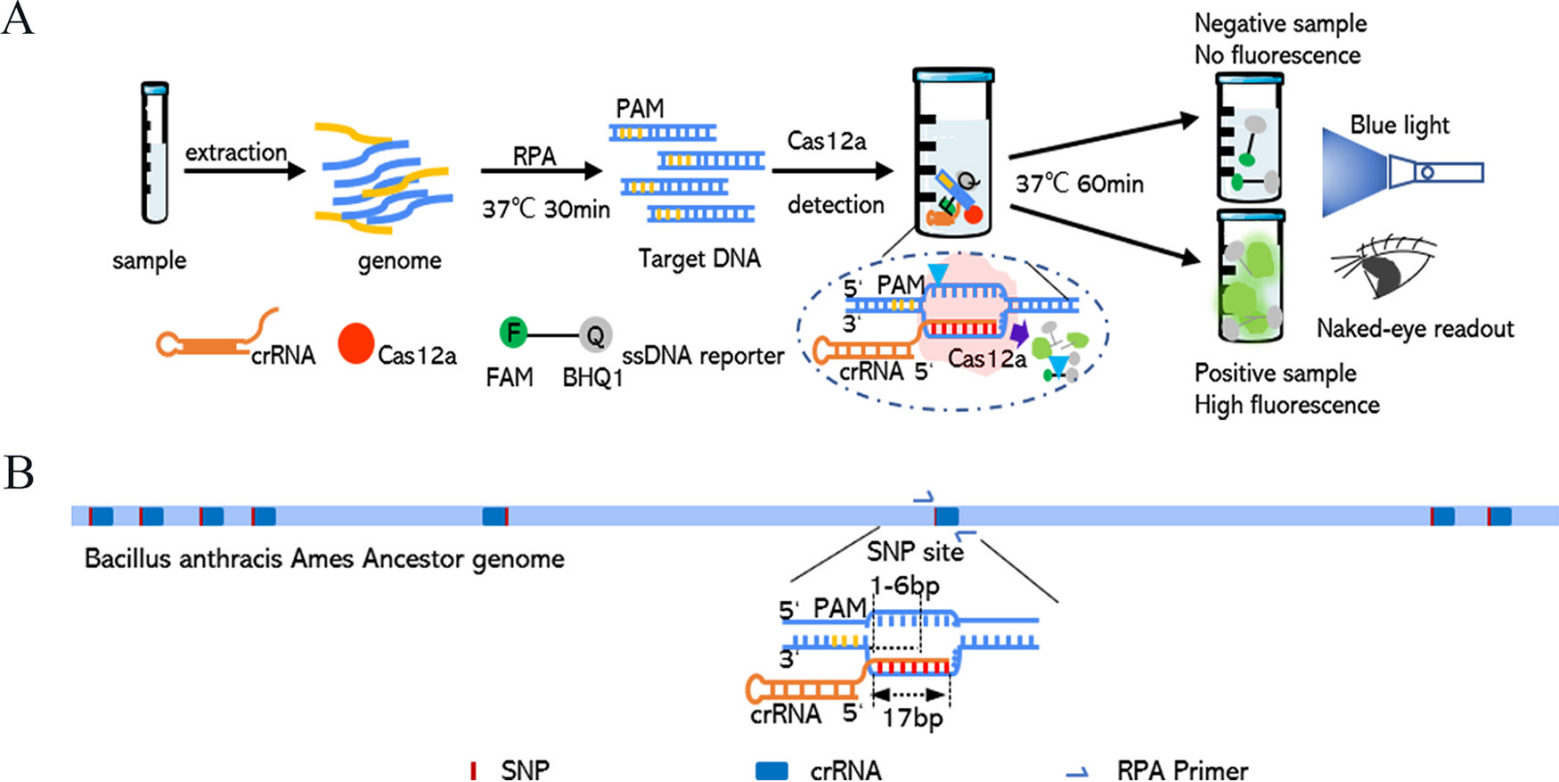
CRISPR/Cas12a-based detection workflow and schematics for B. anthracis detection with a naked-eye readout under blue light. (A) Schematics of the Cas12a-based assay for rapid visual nucleic acid detection. After recombinase polymerase amplification (RPA) for 30 min, the Cas12a enzyme cleavage takes an additional 60 min. The green fluorescent signal of a positive detection of B. anthracis can be observed by the naked eye under blue light with an orange filter. (B) Schematics of the crRNA design for the five novel SNP sites. SNP sites are generally located at bases 1 to 6 downstream of the PAM sequence. The crRNA can target both the coding strand and the noncoding strand.

Having confirmed that the nine SNPs in eProbes_Ba efficiently differentiated B. anthracis from B. cereus and B. thuringiensis *in silico*, we developed a CRISPR/Cas12a-based-detection method with a naked-eye readout under blue light (Fig. 4A). Genomic DNA was extracted from each bacterium and used as the detection substrate. The target gene fragments were amplified within 30 min at 37°C, followed by a CRISPR/Cas12a reaction at 37°C (Fig. 4A). For the specific detection of B. anthracis genomic DNA, CRISPR RNAs (crRNAs) corresponding to the eight novel SNPs on the chromosome of B. anthracis Ames were designed and evaluated in the laboratory (Fig. 4B).

**FIG 5:**
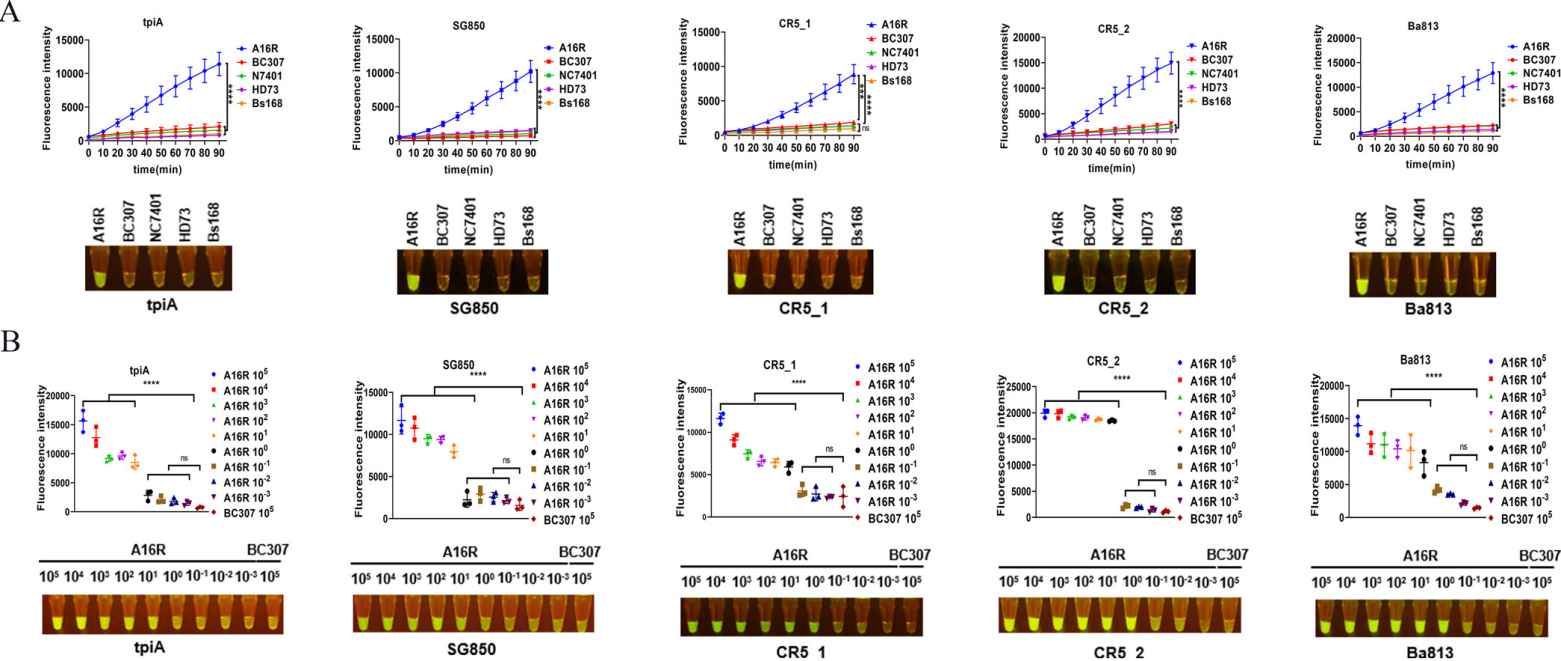
CRISPR/Cas12a-based detection of *Bacillus* spp. with naked-eye readout. (A) These B. anthracis-specific CRISPR RNAs (crRNAs) corresponding to five SNP sites can distinguish B. anthracis (strain A16R) from B. cereus (strains BC307 and NC7401), B. thuringiensis (strain HD73), and B. subtilis (strain Bs168) within 90 min (one-way repeated-measure analysis of variance). Genomic DNA concentrations are shown in Table S9 and other files in the supplemental material. (B) Sensitivity assay using the five crRNAs to detect the B. anthracis strain A16R genomic DNA over a dilution gradient from 5.14 × 10^5^ to 5.14 × 10^−3^ aM (one-way analysis of variance). ****, *P* ≤ 0.0001; ***, *P* ≤ 0.001; *, *P* ≤ 0.05; ns, not significant.

The genomic DNA concentrations of each bacterium was 10^6^ aM (Table S9). Cas12a-mediated detection of each of the five target SNPs produced a robust signal within 60 min when the detection substrate was the product of B. anthracis vaccine strain A16R(pXO1^+^, pXO2^−^) genomic DNA amplification by RPA, which is derived from A16 by exposure to UV radiation (25) (Fig. 5A). Cas12a-mediated detection produced a weak signal within 60 min when the detection substrate was the product of another strain (B. cereus, BC307 and NC7401; B. thuringiensis, HD73; Bacillus subtilis, Bs168) genomic DNA amplifications by RPA. Cas12a-mediated detection allowed B. anthracis to be distinguished from its near-neighbor bacteria by a fluorescent signal. The five target SNPs that can be used to distinguish B. anthracis via RPA are located in the *tpiA*, SG850, CR5_1/2, and Ba813 loci (Fig. 5A). The sensitivity of the CRISPR/Cas12a assay was also determined based on the resulting fluorescence intensity. To determine the detection threshold, genomic DNA of B. anthracis (A16R) was diluted from 5.14 × 10^5^ aM to 5.14 × 10^−3^ aM (Fig. 5B; Table S9). The crRNAs for the target SNPs CR5_2, CR5_1, and Ba813 were the most sensitive and can be used to detect 5 aM of genomic DNA (Fig. 5B). Thus, we chose CR5_2, CR5_1, and Ba813-crRNA for a B. anthracis screening assay. The remaining target loci can be used for confirmatory diagnostic assays.

The other three target SNPs (in *purA*, *pyc*, and *ropB*) can be used to identify B. anthracis via PCR amplification but not via RPA amplification (Fig. S5). In the future, we aim to simultaneously detect multiple SNPs, which would avoid misidentifications due to single SNP mutations, thereby improving the accuracy of the B. anthracis identification.

## DISCUSSION

In previous reports, clinically similar cases of cutaneous anthrax lesions and inhalation anthrax were described as being caused by Bacillus pumilus (26), B. cereus containing B. anthracis toxin genes (27), and B. cereus (28). The characterization of members of the B. cereus group is crucial for decision-making by clinicians and staff at the Centers for Disease Control and Prevention. As described in the introduction, SNPs adopted previously for the identification of B. anthracis have been invalidated as increasingly more genome sequence data have become available (6, 29). Constructing a UPGMA tree is a good way to distinguish strains, but this requires work by skilled bioinformatics professionals.

In this study, we analyzed the genomes of nearly 2,000 B. cereus group strains downloaded from NCBI, identified eight novel SNPs that are characteristic of B. anthracis, and developed software named AGILE that can identify strains based on eProbes (eProbes_Ba and eProbes_Bct). AGILE is a powerful tool for distinguishing B. anthracis from other members of the B. cereus group, which are often not distinguishable by conventional 16S rRNA gene sequencing and BLAST searching. By applying AGILE to validation and test data sets, we discovered 12 non*-*B. anthracis strains that had been assigned as B. anthracis in the NCBI database. The 12 strains have also been found to not belong to the B. anthracis lineage by other international research institutions (30, 31), verifying our results.

With the wider application of whole-genome sequencing in the field of public health, our AGILE tool will become increasingly important: using genome sequence data, AGILE can be used to identify B. anthracis from near-neighbor bacteria *in silico* based on eProbes in seconds. As Fig. 3 shows, 11 isolates from the ISS were unambiguously identified as B. cereus or B. thuringiensis, not B. anthracis, using AGILE. Venkateswaran and colleagues (24) stated that the *gyrB* sequence, DDH and ANI values, and dDDH analysis supported that the 11 ISS isolates were similar to B. anthracis but distant from B. cereus and B. thuringiensis. Phenotypic (motility, positive hemolysis, lack of a capsule, and resistance to gamma phage/penicillin) and genomic (lack of pXO1 and pXO2 plasmids) data were collected, and MLST and whole-genome SNP analyses were performed by them. A large number of the experimental results performed by Venkateswaran et al. provided reasons to exclude the ISS isolates from B. anthracis, which is consistent with the results of the AGILE analysis.

AGILE uses multiple SNP sites; therefore, strains are not missed during the detection process due to poor-quality genomic sequencing. For example, although the nonsense mutation at position 640 in *plcR* proved to be truly unique to B. anthracis, this locus is shortened in several strains of B. cereus and the bases cannot be matched at position 640, which is related to the quality of the sequencing data (Fig. 2A and B). The SNP sites identified in this study are not on virulence plasmids and so are relatively stable. The use of chromosomal molecular markers can identify B. anthracis (pXO1^−^ and/or pXO2^−^) strains, which provides more information for bacterial traceability analysis. Compared with BTyper (32), a command-line tool for classifying B. cereus group isolates via gene detection, AGILE does not involve complicated calculations, processes, or coding operations. It is easy for users to understand the analytical results of AGILE, share data with colleagues, and make definite judgments on the suspected isolates.

Using the SNPs on the chromosome, we have also developed an RPA-Cas12a-based method for the rapid, on-site detection of B. anthracis. The CRISPR5_2, CRISPR5_1, and Ba813 crRNAs can be used to detect B. anthracis with as little as 5 aM genomic DNA. The detection sensitivity is as low as that of HOLMES for detecting the two viruses (19). No precision instruments are required, only a blue light is needed, and detection can be completed in 1.5 h at 37°C.

AGILE based on eProbes_Ba and eProbes_Bct can be used only to identify B. anthracis and distinguish the B. cereus group from other *Bacillus* spp., and other species in the B. cereus group cannot be classified in more detail. However, B. anthracis and B. cereus are common pathogens in this group, and their early and unambiguous diagnostic detection is essential for panic elimination, successful treatment, and disease prevention. We have provided software for use after nucleic acid sequencing and RPA-Cas12a on-site detection for improving the rapid identification of B. anthracis. This paves the way for the development of microbiological diagnostic kits or chips for distinguishing B. anthracis from other B. cereus group members. This technology may aid in rapid responses to anthrax terrorist attacks and help ensure public health security.

## MATERIALS AND METHODS

### Data resources and initial screening of genomes.

Genome sequences of strains were obtained from the GenBank database (https://www.ncbi.nlm.nih.gov/genome/). As shown in Fig. 1, we performed preliminary screening of the downloaded B. cereus group genomic data in local genome database by 16S rRNA gene sequence analysis (33), calculated the average nucleotide identity (ANI) (34, 35) and digital DNA-DNA hybridization (dDDH) (35–37) values, and detected the base at position 640 in the open reading frame (ORF) of the gene *plcR* (13). The dDDH value was attained and analyzed via https://ggdc.dsmz.de/ggdc.php. The other 251 B. anthracis strains from the NCBI database were compared with the genomic sequence of B. anthracis strain Ames Ancestor (GenBank accession no. NC_007530), which is the reference genome for B. anthracis.

### Specific SNP analysis.

As shown in Fig. 1, MLST loci, CRISPR loci, and other loci (*rpoB*, *gyrA*, *gyrB*, Ba813, *plcR*, *ptsI*, *purA*, and SG850) were used to identify species-specific SNPs and thus distinguish among species (see Table S1 in the supplemental material). The sequences of the seven MLST genes can be obtained from the B. cereus MLST website, http://pubmlst.org/bigsdb?db=pubmlst_bcereus_seqdef&page=downloadAlleles&tree=1 (14). We found three CRISPR sites (CRISPR 2, 3, and 5) in B. anthracis strain Ames (GI: 30260195) using the CRISPR finder program (https://crispr.i2bc.paris-saclay.fr/).

The sequences of a selected locus in B. anthracis, B. cereus, and B. thuringiensis were compared. Using local BLAST (blast-2.7.1+), the nucleotide sequence of the selected locus in B. anthracis Ames Ancestor was used as reference for comparison with the sequences of the B. cereus-B. thuringiensis strains. These sequences were imported into MEGA-X software for comparison and analysis to find specific SNP sites that distinguish B. anthracis from its genetic near-neighbors B. cereus and B. thuringiensis (Fig. 1).

### Construction of eProbes.

As shown in Fig. 1, taking the identified SNP in each locus as the base point, 24 bp upstream and 25 bp downstream of the SNP were used to create an electronic probe (eProbe) of 50 bp. A FASTA-format file composed of each eProbe (containing the sequences from all strains) was imported into MEGA software for comparison and analysis. If this tag was identical in all B. anthracis strains, and at least one base in the tag was different from that in all B. cereus and B. thuringiensis strains, we selected it as an eProbe. By adjusting the positions of the upstream and downstream sequences, we identified several B. anthracis*-*specific eProbes (eProbes_Ba) at different loci (Table S2). At the same time, we analyzed the sequences of all B. cereus-B. thuringiensis strains corresponding to the eProbes of B. anthracis and constructed a “degenerate eProbe” for the B. cereus-B. thuringiensis strains (Table S2, eProbes_Bct).

The software was created with Python version 3.7 and employs an *in silico* combination of eProbes_Ba and eProbes_Bct using nucleotide sequencing data. Whether the data to be tested match the probe is used to judge whether the strain is B. anthracis. This does not rely on comparison scoring, and the exact answer is provided directly as output.

### Detection of B. anthracis based on Cas12a-RPA.

The CRISPR/Cas12a system contained the Cas12a protein, the target DNA, B. anthracis-specific CRISPR RNAs (crRNAs), and a single-stranded DNA (ssDNA) reporter. To enable on-site detection, the ssDNA (a 12-base probe) was labeled with 6-carboxyfluorescein (FAM) and the quenching group BHQ1: 5′-FAM–GAGACCGACCTG-3′-BHQ1. When the target DNA of B. anthracis was found by the detection system, the Cas12a/crRNA binary complex formed a ternary complex with the target DNA, and the ssDNA probe was cleaved by Cas12a; the quencher BHQ1 was thus separated from the fluorescent probe FAM, and the resulting green fluorescence could be seen with the naked eye under blue light in the wavelength range of 450 to 480 nm (Fig. 4A). The Cas12a reaction was conducted at 37°C for 60 min (Fig. 4A) in a 20-μL volume (Table S5). Fluorescence intensities were detected at 60 min using a Bio-Rad real-time PCR CFX96 instrument in FAM mode (Life Science, Hercules, CA, USA) or with the naked eye under blue light (Fig. 4A).

The formation of a Cas12a/crRNA/DNA ternary complex requires the target DNA to contain a protospacer-adjacent motif sequence (PAM; a TTTN sequence), which can be added to the primers and introduced during amplification (Fig. 4B). The sequences of the RPA primers used are shown in Table S3, and RPA was performed using the TwistAmp liquid exo kit (TwistDX, Maidenhead, UK). The G+C mol% content of the RPA primers (Table S3) was between 20% and 70%, the melting temperature (*T_m_*) was between 50°C and 100°C, and the RPA reaction was performed at 37°C for 30 min (Table S4). To shorten the reaction time, the length of the DNA product was kept between 100 bp and 150 bp.

The formation of a Cas12a/crRNA/DNA ternary complex requires crRNA. The 17-base cRNA sequence of the target DNA and the universal sequence (5′-AAUUUCUACUGUUGUAGAU-3′) formed a complete crRNA (Table S3) (38). The detection efficiencies of crRNAs for SNP sites located at six different base positions downstream of the PAM were inconsistent. The crRNA targeting the noncoding strand was designed in the same way (Fig. 4B). The sequences of the chosen RPA oligonucleotide primers and crRNAs after preexperimental analysis using PCR (Vazyme catalog no. P510-01; Nanjing, China) are shown in Table S3. The RPA oligonucleotide primers, crRNAs, and single-stranded DNA probes were synthesized by General Biosystems Co. Ltd. (Anhui, China).

## References

[1] Helgason E, Okstad OA, Caugant DA, Johansen HA, Fouet A, Mock M, Hegna I, Kolstø AB. 2000. *Bacillus anthracis*, *Bacillus cereus*, and *Bacillus thuringiensis*–one species on the basis of genetic evidence. Appl Environ Microbiol 66:2627–2630. doi:10.1128/AEM.66.6.2627-2630.2000. 10831447 PMC110590

[2] Reif TC, Johns M, Pillai SD, Carl M. 1994. Identification of capsule-forming *Bacillus anthracis* spores with the PCR and a novel dual-probe hybridization format. Appl Environ Microbiol 60:1622–1625. doi:10.1128/aem.60.5.1622-1625.1994. 8017940 PMC201526

[3] Makino SI, Iinuma-Okada Y, Maruyama T, Ezaki T, Sasakawa C, Yoshikawa M. 1993. Direct detection of *Bacillus anthracis* DNA in animals by polymerase chain reaction. J Clin Microbiol 31:547–551. doi:10.1128/jcm.31.3.547-551.1993. 8458949 PMC262817

[4] Pannucci J, Okinaka RT, Sabin R, Kuske CR. 2002. *Bacillus anthracis* pXO1 plasmid sequence conservation among closely related bacterial species. J Bacteriol 184:134–141. doi:10.1128/JB.184.1.134-141.2002. 11741853 PMC134754

[5] Pannucci J, Okinaka RT, Williams E, Sabin R, Ticknor LO, Kuske CR. 2002. DNA sequence conservation between the *Bacillus anthracis* pXO2 plasmid and genomic sequence from closely related bacteria. BMC Genomics 3:34. doi:10.1186/1471-2164-3-34. 12473162 PMC140023

[6] Ramisse V, Patra G, Vaissaire J, Mock M. 1999. The Ba813 chromosomal DNA sequence effectively traces the whole *Bacillus anthracis* community. J Appl Microbiol 87:224–228. doi:10.1046/j.1365-2672.1999.00874.x. 10475954

[7] Hurtle W, Bode E, Kulesh DA, Kaplan RS, Garrison J, Bridge D, House M, Frye MS, Loveless B, Norwood D. 2004. Detection of the *Bacillus anthracis gyrA* gene by using a minor groove binder probe. J Clin Microbiol 42:179–185. doi:10.1128/JCM.42.1.179-185.2004. 14715750 PMC321681

[8] Yamada S, Ohashi E, Agata N, Venkateswaran K. 1999. Cloning and nucleotide sequence analysis of *gyrB* of *Bacillus cereus*, *B. thuringiensis*, *B. mycoides*, and *B. anthracis* and their application to the detection of *B. cereus* in rice. Appl Environ Microbiol 65:1483–1490. doi:10.1128/AEM.65.4.1483-1490.1999. 10103241 PMC91211

[9] Qi Y, Patra G, Liang X, Williams LE, Rose S, Redkar RJ, Delvecchio VG. 2001. Utilization of the *rpoB* gene as a specific chromosomal marker for real-time PCR detection of *Bacillus anthracis*. Appl Environ Microbiol 67:3720–3727. doi:10.1128/AEM.67.8.3720-3727.2001. 11472954 PMC93078

[10] Daffonchio D, Borin S, Frova G, Gallo R, Mori E, Fani R, Sorlini C. 1999. A randomly amplified polymorphic DNA marker specific for the *Bacillus cereus* group is diagnostic for *Bacillus anthracis*. Appl Environ Microbiol 65:1298–1303. doi:10.1128/AEM.65.3.1298-1303.1999. 10049896 PMC91177

[11] Irenge LM, Durant JF, Tomaso H, Pilo P, Olsen JS, Ramisse V, Mahillon J, Gala JL. 2010. Development and validation of a real-time quantitative PCR assay for rapid identification of *Bacillus anthracis* in environmental samples. Appl Microbiol Biotechnol 88:1179–1192. doi:10.1007/s00253-010-2848-0. 20827474

[12] Faveri JD, Smolowitz RM, Roberts SB. 2009. Development and validation of a real-time quantitative PCR assay for the detection and quantification of Perkinsus marinus in the Eastern oyster, Crassostrea virginica. J Shellfish Res 28:459–464. doi:10.2983/035.028.0306.

[13] Easterday WR, Van Ert MN, Simonson TS, Wagner DM, Kenefic LJ, Allender CJ, Keim P. 2005. Use of single nucleotide polymorphisms in the *plcR* gene for specific identification of *Bacillus anthracis*. J Clin Microbiol 43:1995–1997. doi:10.1128/JCM.43.4.1995-1997.2005. 15815042 PMC1081367

[14] Jolley KA, Maiden MCJ. 2010. BIGSdb: scalable analysis of bacterial genome variation at the population level. BMC Bioinformatics 11:595. doi:10.1186/1471-2105-11-595. 21143983 PMC3004885

[15] Pourcel C, Salvignol G, Vergnaud G. 2005. CRISPR elements in Yersinia pestis acquire new repeats by preferential uptake of bacteriophage DNA, and provide additional tools for evolutionary studies. Microbiology (Reading) 151:653–663. doi:10.1099/mic.0.27437-0. 15758212

[16] Horvath P, Romero DA, Coûté-Monvoisin A-C, Richards M, Deveau H, Moineau S, Boyaval P, Fremaux C, Barrangou R. 2008. Diversity, activity, and evolution of CRISPR loci in *Streptococcus thermophilus*. J Bacteriol 190:1401–1412. doi:10.1128/JB.01415-07. 18065539 PMC2238196

[17] Pardee K, Green AA, Takahashi MK, Braff D, Lambert G, Lee JW, Ferrante T, Ma D, Donghia N, Fan M, Daringer NM, Bosch I, Dudley DM, O’Connor DH, Gehrke L, Collins JJ. 2016. Rapid, low-cost detection of Zika virus using programmable biomolecular components. Cell 165:1255–1266. doi:10.1016/j.cell.2016.04.059. 27160350

[18] Chen JS, Ma E, Harrington LB, Da Costa M, Tian X, Palefsky JM, Doudna JA. 2018. CRISPR-Cas12a target binding unleashes indiscriminate single-stranded DNase activity. Science 360:436–439. doi:10.1126/science.aar6245. 29449511 PMC6628903

[19] Li SY, Cheng QX, Wang JM, Li XY, Zhang ZL, Gao S, Cao RB, Zhao GP, Wang J. 2018. CRISPR-Cas12a-assisted nucleic acid detection. Cell Discov 4:20. doi:10.1038/s41421-018-0028-z. 29707234 PMC5913299

[20] Lobato IM, O’Sullivan CK. 2018. Recombinase polymerase amplification: basics, applications and recent advances. Trends Anal Chem 98:19–35. doi:10.1016/j.trac.2017.10.015. PMC7112910 32287544

[21] Pena-Gonzalez A, Rodriguez-R LM, Marston CK, Gee JE, Gulvik CA, Kolton CB, Saile E, Frace M, Hoffmaster AR, Konstantinidis KT. 2018. Genomic characterization and copy number variation of *Bacillus anthracis* plasmids pXO1 and pXO2 in a historical collection of 412 strains. mSystems 3:e00065-18. doi:10.1128/mSystems.00065-18. 30116789 PMC6093989

[22] Jiménez G, Urdiain M, Cifuentes A, López-López A, Blanch AR, Tamames J, Kämpfer P, Kolstø AB, Ramón D, Martínez JF, Codoñer FM, Rosselló-Móra R. 2013. Description of Bacillus toyonensis sp. nov., a novel species of the Bacillus cereus group, and pairwise genome comparisons of the species of the group by means of ANI calculations. Syst Appl Microbiol 36:383–391. doi:10.1016/j.syapm.2013.04.008. 23791203

[23] Guinebretière M-H, Auger S, Galleron N, Contzen M, De Sarrau B, De Buyser M-L, Lamberet G, Fagerlund A, Granum PE, Lereclus D, De Vos P, Nguyen-The C, Sorokin A. 2013. Bacillus cytotoxicus sp. nov. is a novel thermotolerant species of the Bacillus cereus group occasionally associated with food poisoning. Int J Syst Evol Microbiol 63:31–40. doi:10.1099/ijs.0.030627-0. 22328607

[24] Venkateswaran K, Singh NK, Checinska Sielaff A, Pope RK, Bergman NH, van Tongeren SP, Patel NB, Lawson PA, Satomi M, Williamson CHD, Sahl JW, Keim P, Pierson D, Perry J. 2017. Non-toxin-producing *Bacillus cereus* strains belonging to the *B. anthracis* clade isolated from the International Space Station. mSystems 2:e00021-17. doi:10.1128/mSystems.00021-17. 28680972 PMC5487513

[25] Liu X, Wang D, Ren J, Tong C, Feng E, Wang X, Zhu L, Wang H. 2013. Identification of the immunogenic spore and vegetative proteins of *Bacillus anthracis* vaccine strain A16R. PLoS One 8:e57959. doi:10.1371/journal.pone.0057959. 23516421 PMC3596338

[26] Tena D, Martinez-Torres JA, Perez-Pomata MT, Sáez-Nieto JA, Rubio V, Bisquert J. 2007. Cutaneous infection due to Bacillus pumilus: report of 3 cases. Clin Infect Dis 44:e40–e42. doi:10.1086/511077. 17243047

[27] Avashia SB, Riggins WS, Lindley C, Hoffmaster A, Drumgoole R, Nekomoto T, Jackson PJ, Hill KK, Williams K, Lehman L, Libal MC, Wilkins PP, Alexander J, Tvaryanas A, Betz T. 2007. Fatal pneumonia among metalworkers due to inhalation exposure to Bacillus cereus containing *Bacillus anthracis* toxin genes. Clin Infect Dis 44:414–416. doi:10.1086/510429. 17205450

[28] Saikia L, Gogoi N, Das PP, Sarmah A, Punam K, Mahanta B, Bora S, Bora R. 2019. Bacillus cereus-attributable primary cutaneous anthrax-like infection in newborn infants, India. Emerg Infect Dis 25:1261–1270. doi:10.3201/eid2507.181493. 31211665 PMC6590766

[29] Keim P, Price LB, Klevytska AM, Smith KL, Schupp JM, Okinaka R, Jackson PJ, Hugh-Jones ME. 2000. Multiple-locus variable-number tandem repeat analysis reveals genetic relationships within *Bacillus anthracis*. J Bacteriol 182:2928–2936. doi:10.1128/JB.182.10.2928-2936.2000. 10781564 PMC102004

[30] Pisarenko SV, Eremenko EI, Ryazanova AG, Kovalev DA, Buravtseva NP, Aksenova LY, Evchenko AY, Semenova OV, Bobrisheva OV, Kuznetsova IV, Golovinskaya TM, Tchmerenko DK, Kulichenko AN, Morozov VY. 2019. Genotyping and phylogenetic location of one clinical isolate of *Bacillus anthracis* isolated from a human in Russia. BMC Microbiol 19:165. doi:10.1186/s12866-019-1542-3. 31315564 PMC6637652

[31] Abdel-Glil MY, Chiaverini A, Garofolo G, Fasanella A, Parisi A, Harmsen D, Jolley KA, Elschner MC, Tomaso H, Linde J, Galante D. 2021. A whole-genome-based gene-by-gene typing system for standardized high-resolution strain typing of *Bacillus anthracis*. J Clin Microbiol 59:e02889-20. doi:10.1128/JCM.02889-20. 33827898 PMC8218748

[32] Carroll LM, Kovac J, Miller RA, Wiedmann M. 2017. Rapid, high-throughput identification of anthrax-causing and emetic *Bacillus cereus* group genome assemblies via BTyper, a computational tool for virulence-based classification of *Bacillus cereus* group isolates by using nucleotide sequencing data. Appl Environ Microbiol 83:e01096-17. doi:10.1128/AEM.01096-17. 28625989 PMC5561296

[33] Stackebrandt E, Goebel BM. 1994. Taxonomic note: a place for DNA-DNA reassociation and 16S rRNA sequence analysis in the present species definition in bacteriology. Int J Syst Bacteriol 44:846–849. doi:10.1099/00207713-44-4-846.

[34] Liu Y, Du J, Lai Q, Zeng R, Ye D, Xu J, Shao Z. 2017. Proposal of nine novel species of the Bacillus cereus group. Int J Syst Evol Microbiol 67:2499–2508. doi:10.1099/ijsem.0.001821. 28792367

[35] Chun J, Oren A, Ventosa A, Christensen H, Arahal DR, da Costa MS, Rooney AP, Yi H, Xu X-W, De Meyer S, Trujillo ME. 2018. Proposed minimal standards for the use of genome data for the taxonomy of prokaryotes. Int J Syst Evol Microbiol 68:461–466. doi:10.1099/ijsem.0.002516. 29292687

[36] Goris J, Konstantinidis KT, Klappenbach JA, Coenye T, Vandamme P, Tiedje JM. 2007. DNA-DNA hybridization values and their relationship to whole-genome sequence similarities. Int J Syst Evol Microbiol 57:81–91. doi:10.1099/ijs.0.64483-0. 17220447

[37] Richter M, Rosselló-Móra R. 2009. Shifting the genomic gold standard for the prokaryotic species definition. Proc Natl Acad Sci USA 106:19126–19131. doi:10.1073/pnas.0906412106. 19855009 PMC2776425

[38] Gao Z, Herrera-Carrillo E, Berkhout B. 2018. Improvement of the CRISPR-Cpf1 system with ribozyme-processed crRNA. RNA Biol 15:1458–1467. doi:10.1080/15476286.2018.1551703. 30470168 PMC6333430

