# Supplementary material for: Rapid Identification of Bacillus anthracis In Silico and On-Site Using Novel Single-Nucleotide Polymorphisms
Source: Microbiol Spectr. 2022 May 16;10(3):e02285-21. doi: 10.1128/spectrum.02285-21 (PMC9241702; doi:10.1128/spectrum.02285-21)
Supplement: SUPPLEMENTAL FILE 1 — Supplemental material. Download spectrum.02285-21-s001.pdf, PDF file, 3.6 MB [file spectrum.02285-21-s001.pdf]

Appendix

Fig. S1 Alignment analysis of four loci (*gmk*, *gyrB*, *pta*, *pur*) of *B. anthracis*, *B. cereus*, and *B. thuringiensis* with the reference strain *B. anthracis* Ames Ancestor

Fig. S2 Screenshot of SNP screening for the *glpF* locus

Fig. S3 Screenshot of SNP screening for the nine loci

Fig. S4 Screenshot of AGILE V1.1 software analysis (Anthracis Genome-based Identification with high-fideLity E-probe) for identification of *B. anthracis*

Fig. S5 Three target SNPs (in *purA*, *pyc*, *ropB*) can be used to identify *B. anthracis* via PCR amplification with Cas12a

Fig. S6 The website screenshot of Github.

Fig. S7 The website screenshot of decompressing.

Fig. S8 Workflow of the Select File model.

Fig. S9 Workflow of the Select Folder model.

Table S1 Selected loci in this study.

Table S2 Information on two eProbe sets used in this study

Table S3 Target sequences, RPA primers, and crRNAs used for detection in this study

Table S4 RPA reaction conditions (50 μL)

Table S5 Cas12a reaction conditions (20 μL)

Table S6 Information on *Bacillus* species (4947 strains)

Table S7 Assembly ID and renaming of *Bacillus anthracis* in this study

Table S8 Assembly ID and renaming of the representative strains of *B. cereus sensu lato*

Table S9 Genomic DNA concentration

Specific SNP analysis of loci

Considering the 12 eliminated *B. anthracis* strains, the nucleotide sequences of these loci in the 240 putative *B. anthracis* strains (*n* = 1980) were analyzed by local BLAST searches using blast-2.7.1+.

First, the sequences of 18 loci were found in the reference strain (*Bacillus anthracis* Ames_Ancestor) and converted into fasta format. The nucleotide sequences of 18 loci of *B. anthracis* Ames_Ancestor were compared among BA, BC, and BT (hereinafter referred to as Bct); the completely homologous loci were eliminated, and suitable loci were initially screened for subsequent use. The nucleotide sequences of the remaining loci were individually truncated in all strains to construct aligned sequences (fasta format).

These sequences were imported into mega software (MEGA-X) for comparison and analysis to find specific SNP sites that can be used to distinguish *B. anthracis* from its close source strains (Bct), and we determined the positions of these SNP sites in the reference sequence.

In Fig. S1, the four loci (*gmk*, *gyrB*, *pta*, *pur*) were completely homologous among *B. anthracis*, *B. cereus,* and *B. thuringiensis* and were eliminated from the locus set after alignment (N = 14). Further analysis found that six loci (crispR2, crispR3, *glpF*, *gyrA*, *ilvD,* and *ptsI*) had no specific SNPs in *B. anthracis* that could be used to distinguish it from the Bct strain and were also eliminated (N = 8). As Fig. S2 shows (we selected *glpF* as an example to show the results in screenshots), the situation for other loci was the same. As shown in Fig. S3, specific SNP sites that could be used to distinguish *B. anthracis* from Bct strains were identified in eight loci (one in each of Ba813, *plcR*, *purA*, *pyc*, *rpoB*, SG850, and *tpiA*, and two in CRISPR5).

A

Site	Query	SubjId	Identica	AlignLengt	Misma	Gap	Query	QueryEnd	SubjStart	SubjEnd	ExpectVa	BitScore
**gmk**	BA001_Ames_Ancestor	BA001_Ames_Ancestor	**100**	618	0	0	1	618	3688756	3688139	0	1142
**gmk**	BA001_Ames_Ancestor	BA002_Ames	**100**	618	0	0	1	618	3688629	3688012	0	1142
**gmk**	BA001_Ames_Ancestor	BA003_Sterne	**100**	618	0	0	1	618	3689324	3688707	0	1142
**gmk**	BA001_Ames_Ancestor	BA252_ANSES_90_contig36	**100**	618	0	0	1	618	226773	226156	0	1142
**gmk**	BA001_Ames_Ancestor	BC0034_ISSFR-9F	**100**	618	0	0	1	618	3642589	3641972	0	1142
**gmk**	BA001_Ames_Ancestor	BC0035_ISSFR-3F	**100**	618	0	0	1	618	1616214	1616831	0	1142
**gmk**	BA001_Ames_Ancestor	BC0036_JEM-2	**100**	618	0	0	1	618	3231189	3231806	0	1142
**gmk**	BA001_Ames_Ancestor	BC0052_MH19	**100**	618	0	0	1	618	3710607	3709990	0	1142
**gmk**	BA001_Ames_Ancestor	BC0322_ISSFR-23F_contig12	**100**	618	0	0	1	618	337281	337898	0	1142
**gmk**	BA001_Ames_Ancestor	BC0343_MOD1_Bc88_contig29	**100**	618	0	0	1	618	49318	49935	0	1142
**gmk**	BA001_Ames_Ancestor	BC0384_MOD1_Bc214_contig22	**100**	618	0	0	1	618	158009	157392	0	1142
**gmk**	BA001_Ames_Ancestor	BC0982_MYb220_contig5	**100**	618	0	0	1	618	68140	68757	0	1142
**gmk**	BA001_Ames_Ancestor	BC0983_MYb212_contig33	**100**	618	0	0	1	618	173242	172625	0	1142
**gmk**	BA001_Ames_Ancestor	BC0999_MH19_contig0	**100**	618	0	0	1	618	174132	173515	0	1142
**gmk**	BA001_Ames_Ancestor	BC1048_BIOML-A1_contig32	**100**	618	0	0	1	618	68592	69209	0	1142
**gmk**	BA001_Ames_Ancestor	BC1112_16-00195_contig42	**100**	618	0	0	1	618	67972	68589	0	1142
**gmk**	BA001_Ames_Ancestor	BT001_97-27	**100**	618	0	0	1	618	3710303	3709686	0	1142
**gmk**	BA001_Ames_Ancestor	BT020_97-27	**100**	618	0	0	1	618	3959753	3960370	0	1142
**gmk**	BA001_Ames_Ancestor	BT058_FDAARGOS_792	**100**	618	0	0	1	618	2911777	2911160	0	1142
**gmk**	BA001_Ames_Ancestor	BT141_BGSC_4AY1_contig69	**100**	618	0	0	1	618	977478	978095	0	1142
**gmk**	BA001_Ames_Ancestor	BT543_4XX3_contig732	**100**	618	0	0	1	618	674619	675236	0	1142
**gmk**	BA001_Ames_Ancestor	BT545_4XX2_contig128	**100**	618	0	0	1	618	674537	675154	0	1142
**gmk**	BA001_Ames_Ancestor	BT546_4XX1_contig86	**100**	618	0	0	1	618	169521	168904	0	1142

B

Site	Query	SubjId	Identical%	AlignLengt	Mismatch	Gap	QueryStart	QueryEnd	SubjStart	SubjEnd	ExpectVal	BitScore
**pta**	BA001_Ames_Ancestor	BA001_Ames_Ancestor	**100**	972	0	0	1	972	5123262	5122291	0	1796
**pta**	BA001_Ames_Ancestor	BA002_Ames	**100**	972	0	0	1	972	5123136	5122165	0	1796
**pta**	BA001_Ames_Ancestor	BA003_Sterne	**100**	972	0	0	1	972	5124507	5123536	0	1796
**pta**	BA001_Ames_Ancestor	BA004_CDC_684	**100**	972	0	0	1	972	5125833	5124862	0	1796
**pta**	BA001_Ames_Ancestor	BA250_ANSES_32_contig11	**100**	972	0	0	1	972	28940	27969	0	1796
**pta**	BA001_Ames_Ancestor	BA251_ANSES_88_contig17	**100**	972	0	0	1	972	28950	27979	0	1796
**pta**	BA001_Ames_Ancestor	BA252_ANSES_90_contig31	**100**	972	0	0	1	972	28605	27634	0	1796
**pta**	BA001_Ames_Ancestor	BC0034_ISSFR-9F	**100**	972	0	0	1	972	5044666	5043695	0	1796
**pta**	BA001_Ames_Ancestor	BC0035_ISSFR-3F	**100**	972	0	0	1	972	214098	215069	0	1796
**pta**	BA001_Ames_Ancestor	BC0036_JEM-2	**100**	972	0	0	1	972	1829072	1830043	0	1796
**pta**	BA001_Ames_Ancestor	BC0233_JRS4_contig379	**100**	972	0	0	1	972	73	1044	0	1796
**pta**	BA001_Ames_Ancestor	BC0322_ISSFR-23F_contig11	**100**	972	0	0	1	972	1084134	1083163	0	1796

C

Site	Query	SubjId	Identical%	AlignLengt	Mismatch	Gap	QueryStart	QueryEnd	SubjStart	SubjEnd	ExpectVal	BitScore
**purH(pur)**	BA001_Ames_Ancestor	BA001_Ames_Ancestor	**100**	1536	0	0	1	1536	305573	307108	0	2837
**purH(pur)**	BA001_Ames_Ancestor	BA002_Ames	**100**	1536	0	0	1	1536	305573	307108	0	2837
**purH(pur)**	BA001_Ames_Ancestor	BA003_Sterne	**100**	1536	0	0	1	1536	305586	307121	0	2837
**purH(pur)**	BA001_Ames_Ancestor	BA250_ANSES_32_contig19	**100**	1536	0	0	1	1536	110803	109268	0	2837
**purH(pur)**	BA001_Ames_Ancestor	BA251_ANSES_88_contig5	**100**	1536	0	0	1	1536	10806	12341	0	2837
**purH(pur)**	BA001_Ames_Ancestor	BA252_ANSES_90_contig21	**100**	1536	0	0	1	1536	10778	12313	0	2837
**purH(pur)**	BA001_Ames_Ancestor	BC0008_AH820	**100**	1536	0	0	1	1536	313342	314877	0	2837
**purH(pur)**	BA001_Ames_Ancestor	BC0020_3a	**100**	1536	0	0	1	1536	3320197	3318662	0	2837
**purH(pur)**	BA001_Ames_Ancestor	BC0025_S2-8	**100**	1536	0	0	1	1536	3865209	3863674	0	2837
**purH(pur)**	BA001_Ames_Ancestor	BC0067_FDAARGOS_781	**100**	1536	0	0	1	1536	5101937	5103472	0	2837
**purH(pur)**	BA001_Ames_Ancestor	BC0068_FDAARGOS_780	**100**	1536	0	0	1	1536	2847032	2845497	0	2837
**purH(pur)**	BA001_Ames_Ancestor	BC0083_95_8201_contig0	**100**	1536	0	0	1	1536	365637	364102	0	2837
**purH(pur)**	BA001_Ames_Ancestor	BC0233_JRS4_contig70	**100**	1536	0	0	1	1536	11989	10454	0	2837
**purH(pur)**	BA001_Ames_Ancestor	BC0235_PE8-121b_contig209	**100**	1536	0	0	1	1536	24331	22796	0	2837
**purH(pur)**	BA001_Ames_Ancestor	BT017_HD1011	**100**	1536	0	0	1	1536	1506067	1507602	0	2837
**purH(pur)**	BA001_Ames_Ancestor	BT019_HD682	**100**	1536	0	0	1	1536	4435077	4433542	0	2837
**purH(pur)**	BA001_Ames_Ancestor	BT055_FDAARGOS_795	**100**	1536	0	0	1	1536	2388871	2390406	0	2837
**purH(pur)**	BA001_Ames_Ancestor	BT057_FDAARGOS_794	**100**	1536	0	0	1	1536	4618835	4617300	0	2837
**purH(pur)**	BA001_Ames_Ancestor	BT069_BGSC_4BA1_contig0	**100**	1536	0	0	1	1536	266361	267896	0	2837
**purH(pur)**	BA001_Ames_Ancestor	BT142_BGSC_4BA1_contig18	**100**	1536	0	0	1	1536	10929	12464	0	2837

D

Site	Query	SubjId	Identical%	AlignLengt	Mismatch	Gap	QueryStart	QueryEnd	SubjStart	SubjEnd	ExpectVal	BitScore
**gyrB**	BA001_Ames_Ancestor	BA001_Ames_Ancestor	**100**	1923	0	0	1	1923	4584	6506	0	3552
**gyrB**	BA001_Ames_Ancestor	BA002_Ames	**100**	1923	0	0	1	1923	4584	6506	0	3552
**gyrB**	BA001_Ames_Ancestor	BA003_Sterne	**100**	1923	0	0	1	1923	4585	6507	0	3552
**gyrB**	BA001_Ames_Ancestor	BA004_CDC_684	**100**	1923	0	0	1	1923	4458	6380	0	3552
**gyrB**	BA001_Ames_Ancestor	BA252_ANSES_90_contig75	**100**	1923	0	0	1	1923	4678	2756	0	3552
**gyrB**	BA001_Ames_Ancestor	BC0008_AH820	**100**	1923	0	0	1	1923	4585	6507	0	3552
**gyrB**	BA001_Ames_Ancestor	BC0034_ISSFR-9F	**100**	1923	0	0	1	1923	5153460	5155382	0	3552
**gyrB**	BA001_Ames_Ancestor	BC0035_ISSFR-3F	**100**	1923	0	0	1	1923	105304	103382	0	3552
**gyrB**	BA001_Ames_Ancestor	BC0036_JEM-2	**100**	1923	0	0	1	1923	1720278	1718356	0	3552
**gyrB**	BA001_Ames_Ancestor	BC0099_W_contig87	**100**	1923	0	0	1	1923	5454	3532	0	3552
**gyrB**	BA001_Ames_Ancestor	BC0101_NVH0597-99_contig46	**100**	1923	0	0	1	1923	26138	24216	0	3552
**gyrB**	BA001_Ames_Ancestor	BC0124_ISP3191_contig1	**100**	1923	0	0	1	1923	80674	78752	0	3552
**gyrB**	BA001_Ames_Ancestor	BC0322_ISSFR-23F_contig11	**100**	1923	0	0	1	1923	1192918	1194840	0	3552
**gyrB**	BA001_Ames_Ancestor	BC0344_MOD1_Bc122_contig1	**100**	1923	0	0	1	1923	5032	3110	0	3552
**gyrB**	BA001_Ames_Ancestor	BC1030_DE0595_contig7	**100**	1923	0	0	1	1923	107732	109654	0	3552
**gyrB**	BA001_Ames_Ancestor	BT066_BGSC_4AJ1_contig0	**100**	1923	0	0	1	1923	5172945	5171023	0	3552
**gyrB**	BA001_Ames_Ancestor	BT110_BGSC_4AC1_contig97	**100**	1923	0	0	1	1923	4703	2781	0	3552
**gyrB**	BA001_Ames_Ancestor	BT119_BGSC_4AJ1_contig43	**100**	1923	0	0	1	1923	4872	2950	0	3552
**gyrB**	BA001_Ames_Ancestor	BT141_BGSC_4AY1_contig58	**100**	1923	0	0	1	1923	4905	2983	0	3552
**gyrB**	BA001_Ames_Ancestor	BT470_BGSC_4AJ1_contig33	**100**	1923	0	0	1	1923	4667	2745	0	3552
**gyrB**	BA001_Ames_Ancestor	BT496_G25-42_contig196	**100**	1923	0	0	1	1923	4715	2793	0	3552
**gyrB**	BA001_Ames_Ancestor	BT543_4XX3_contig833	**100**	1923	0	0	1	1923	543646	545568	0	3552
**gyrB**	BA001_Ames_Ancestor	BT545_4XX2_contig97	**100**	1923	0	0	1	1923	137350	139272	0	3552
**gyrB**	BA001_Ames_Ancestor	BT546_4XX1_contig115	**100**	1923	0	0	1	1923	145830	147752	0	3552

Fig. S1 Alignment analysis of four loci (*gmk*, *gyrB*, *pta*, *pur*) of *B. anthracis*, *B. cereus,*

and *B. thuringiensis* with the reference strain *B. anthracis* Ames Ancestor. The four loci are completely homologous between *B. anthracis*, *B. cereus,* and *B. thuringiensis*. *B. cereus* and *B. thuringiensis* are highlighted in green. The identity values are marked in yellow.

glpF

```
#MEGA
!Title Phylogenetic Analysis;
!Format
   DataType=Nucleotide
   NSeqs=338 NSites=262
   Identical=. Missing=? Indel=-;
!Domain=Data;
[                                           111 1111111111 1111111111 1111111111 1222222222 2222222222 ]
[                        111222333 4444555666 7788999000 1112222333 4444555566 6677888999 9000011112 2333333444 ]
[                       7138125369 0568147069 2547069258 2470139568 1478036912 5817039258 9145701362 9123489023 ]
#BA001_Ames_Ancestor_N194 GTTAGTATGG TCGATTCCTT TAGGGGATTT ATTTAACCTA GAGGTAGCTG TAATGAGTAT TGGAAATAAG TCCCAGGTAC
#BA004_CDC_684_N14        .......... .......... .......... .......... .......... .......... .......... ..........
#BA007_BF1_N18            .......... .......... .......... .......... .......... .......... .......... ..........
#BA013_2000031021_N6      .......... .......... .......... .......... ........C. .......... .......... ..........
#BA016_Han_N1             .......... .......... .......... .......... .......... ....-..... .......... ..........
#BA062_A2012_N1           ---------- ---------. .......... .......... .......... .......... .......... ..........
#BA139_3631_1C_N1         .......... .......... .......... .......... .......... .......... .......... ..........
#BC0001_ATCC14579_N63     .......... .......... ...A....A. .......... A......TC. .......... .A........ .....A.C..
#BC0002_ATCC10987_N6      .......... .......... .......... .......... A.....ATC. .......... .A........ .....A....
#BC0003_E33L_N2           .......... .......... .......... .......... A......TCA .......... .A........ .....A....
#BC0004_Q1_N70            .......... .......... .......... .......... .......TC. .......... .......... .....A....
#BC0005_B4264_N3          .......... .......... ...A....A. .......... A......TC. .......... .A........ .....A....
#BC0007_G9842_N133        .......... .......... ...A....A. .......... A......TC. .......... .A....C... .....A....
#BC0008_AH820_N2          .......... .......... G......... .......... ........C. .......... .......... ..........
#BC0009_03BB102_N14       .......... .......... .......... .......... .......TC. .......... .......... .....A....
#BC0011_F837_76_N5        .......... .......... .......... .......... .......TC. .......... .A....C... .....A....
#BC0013_FRI-35_N7         .......... .......... .......... .......... A.....ATC. .......... .A........ .....A....
#BC0014_A1_N2             .......... .......... .......... .......... A......TC. .......... .AA....... .....A...T
#BC0015_FT9_N1            .......... .......... ...A..G... ..C....... ......ATC. .......... .A........ .....A....
#BC0016_03BB87_N7         .......... ...T...... .......... ..A....... ......ATC. .......... .A........ .....A....
#BC0017_D17_N4            .......... .......... .......... .......... .......TC. .......... .......... .....A....
#BC0020_3a_N7             .......... .......... G......... .......... ........C. .......... .......... ..........
#BC0022_ATCC4342_N3       .......... .......... ...A..G... ..C....... ......ATC. .......... .A........ .....A....
```

Fig. S2 Screenshot of SNP screening at the *glpF* locus. There are no specific SNP sites in the *B. anthracis glpF* locus that can be used to distinguish it from closely related Bct strains. Because there were too many strains, only the alignment results of *glpF* for selected strains are displayed.

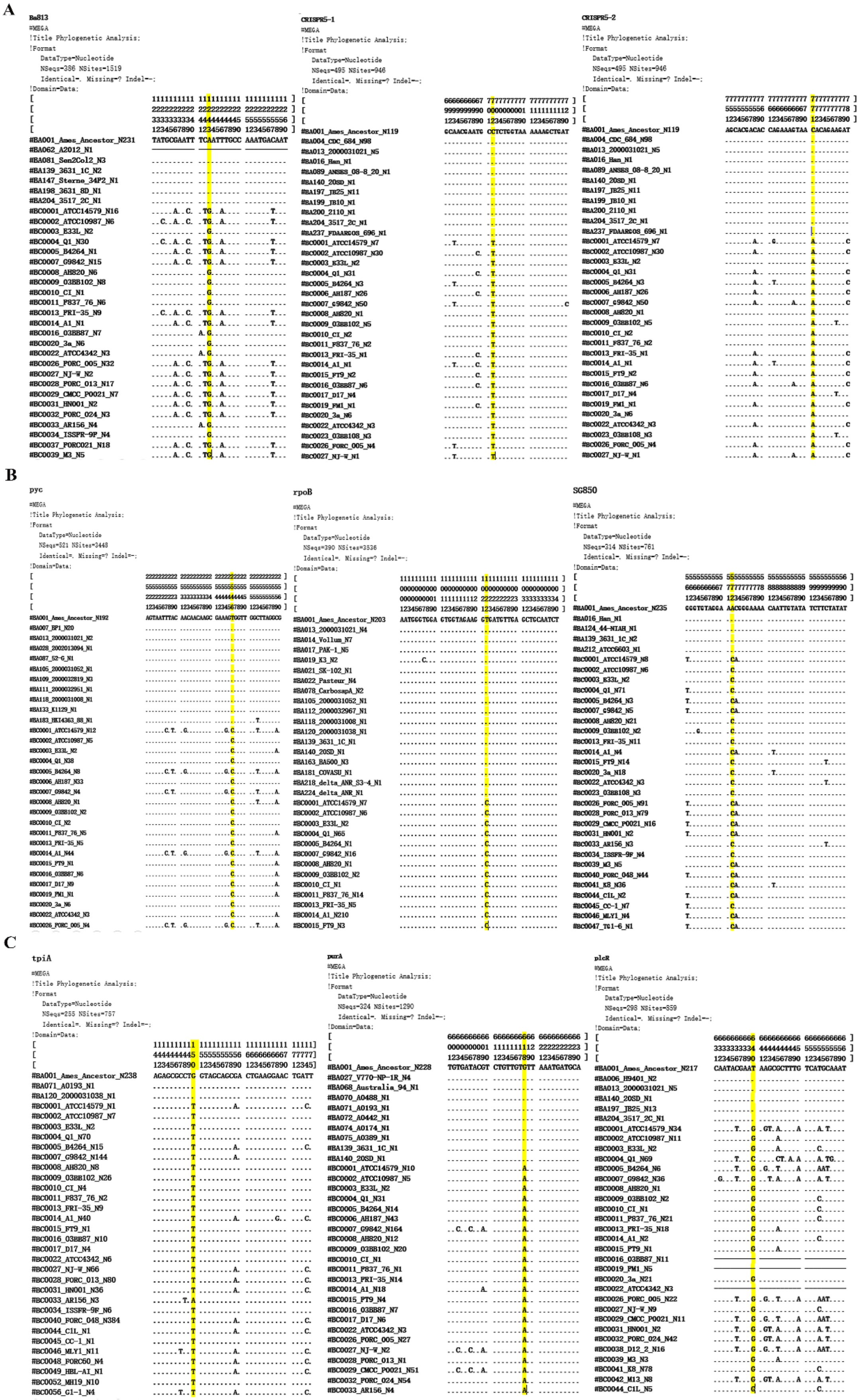

Fig. S3 Screenshot of SNP screening at the nine loci. Specific SNP sites that could be

used to distinguish *B. anthracis* from Bct strains were identified in eight loci (one in each of Ba813, *plcR*, *purA*, *pyc*, *rpoB*, SG850, and *tpiA*, and two in CRISPR5). Because there were too many strains, only the alignment results of nine loci from selected strains are displayed. The SNP sites are marked in yellow.

Screening of 12 doubtful *B. anthracis* strains using AGILE

After screening with AGILE V1.1, the 12 doubtful *B. anthracis* strains and BC307 and NC7401 were revealed to be *B. cereus*

Operation Results

	Strains	Ba813_Ba	crispR5_Ba1	crispR5_Ba2	plcR_Ba	purA_Ba	pyc_Ba	rpoB_Ba	SG850_Ba	tpiA_Ba	Ba813_Bct	crispR5_Bct1	crispR5_Bct2	plcR_Bct	purA_Bct	pyc_Bct	rpoB_Bct	SG850_Bct	tpiA_Bct	Is_B.anthracis
1	Ab_BA052_MCCC_1A02161.fna	-	-	-	-	-	-	-	-	-	G	T	A	G	A	C	C	C	T	No
2	Ab_BA053_MCCC_1A01412.fna	-	-	-	-	-	-	-	-	-	G	T	A	G	A	C	C	C	A	No
3	Ab_BA126_RIT375.fna	-	-	-	-	-	-	-	-	-	G	T	A	G	A	C	C	C	T	No
4	Ab_BA143_PFAB2.fna	-	-	-	-	-	-	-	-	-	G	T	A	G	A	C	C	C	T	No
5	Ab_BA145_N1ZF-2.fna	-	-	-	-	-	-	-	-	-	G	T	A	G	A	C	C	C	T	No
6	Ab_BA146_L19.fna	-	-	-	-	-	-	-	-	-	G	T	A	G	A	C	C	C	A	No
7	Ab_BA150_F34.fna	-	-	-	-	-	-	-	-	-	-	T	A	G	A	C	C	C	T	No
8	Ab_BA168_AFS095574.fna	-	-	-	-	-	-	-	-	-	G	T	A	-	A	C	C	C	A	No
9	Ab_BA169_AFS081271.fna	-	-	-	-	-	-	-	-	-	G	T	-	-	A	C	C	C	A	No
10	Ab_BA170_AFS072084.fna	-	-	-	-	-	-	-	-	-	G	T	A	G	A	C	C	C	T	No
11	Ab_BA171_AFS057383.fna	-	-	-	-	-	-	-	-	-	G	T	A	-	A	C	C	C	A	No
12	Ab_BA172_AFS029941.fna	-	-	-	-	-	-	-	-	-	G	T	A	C	A	C	C	C	T	No
13	BA001_Ames_Ancestor.fna	A	C	C	T	G	T	T	A	G	-	-	-	-	-	-	-	-	-	Yes
14	BC0001_ATCC14579.fna	-	-	-	-	-	-	-	-	-	G	T	A	G	A	C	C	C	T	No
15	BC0012_NC7401.fna	-	-	-	-	-	-	-	-	-	G	T	A	C	A	C	C	C	T	No
16	BC307.fna	-	-	-	-	-	-	-	-	-	G	T	A	C	A	C	C	C	T	No
17	BT001_97-27.fna	-	-	-	-	-	-	-	-	-	G	T	A	G	A	C	C	C	T	No

Fig. S4 Screenshot of Anthracis Genome-based Identification with high-fideLity E-probe (AGILE) V1.1 software analysis for identification of *B. anthracis*. "–": no bases could be detected at this site.

These crRNAs corresponding to the three SNP sites (in *purA, ropB, pyc* loci) can distinguish *B. anthracis* (A16R) from *B. cereus* (BC307 and NC7401) via PCR-Cas12a.

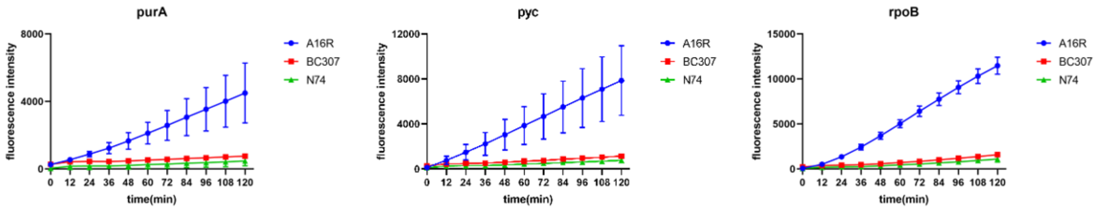

Fig. S5 The other three target SNPs (in *purA*, *pyc*, *ropB*) can be used to identify *B. anthracis* via PCR amplification with Cas12a.

Table S1 The selected loci in this study.

Gene	Start	End	Length(bp)	strand	PID	Synonym	Product
glpF	1014674	1015495	822	+	47526304	GBAA1025	glycerol uptake facilitator protein
gmk	3688139	3688756	618	-	47529302	GBAA4009	guanylate kinase
ilvD	1735111	1736784	1674	+	47527144	GBAA1853	dihydroxy-acid dehydratase
pta	5122291	5123262	972	-	47530962	GBAA5636	phosphate acetyltransferase
purH	305573	307108	1536	+	47525561	GBAA0298	bifunctional phosphoribosylaminoi-midazolecarboxamide formyltransferase/IMP cyclohydrolase
pyc	3809107	3812553	3447	-	47529455	GBAA4157	pyruvate carboxylase
tpiA	4861362	4862117	756	-	47778395	GBAA5366	triosephosphate isomerase
gyrA	6595	9066	2472	+	47525260	GBAA0006	DNA gyrase subunit A
gyrB	4584	6506	1923	+	47525259	GBAA0005	DNA topoisomerase IV subunit B
rpoB	108391	111924	3534	+	50196908	GBAA0102	DNA-directed RNA polymerase beta subunit
SG850	1491267	1493204	1938	+	47526861	GBAA1586	hypothetical protein
ptsI	3901931	3903643	1713	-	47529560	GBAA4267	phosphoenolpyruvate-protein phosphotransferase
Ba813	4563990	4565507	1518	-	47530330	GBAA5031	hypothetical protein
CrispR2	1176218	1177326	1109	+	-	-	-
CrispR3	2719536	2720733	1198	+	-	-	-
CrispR5	5014835	5015740	906	-	47530842	GBAA5524	stage ii sporulation protein
plcR	5081211	5082068	858	-	-	GBAA5595	transcriptional regulator PlcR,putative
purA	5207311	5208600	1290	-	47531053	GBAA5716	adenylosuccinate synthetase

Table S2 The information of two eProbe sets in this study.

Locus	Base change	eProbes_Ba	eProbes_Bct
Ba813	A-G	CCATTGCTAATGTATGCGAATTTC**A**ATTTGCCAAATGACAATTTAGGTTT	[CG]C[GAT][CT]T[AG][TC]TAAT[GAC][TC]A[TC][GA]C[AG]AA[CT]T[TA][TC]**G**A[TC][ATCG]T[GA][CA]C[AG]AATGA[TC][AG]A[TC][TAC]T[AG]GG[TCGA][TG]T
crispR5_1	C-T	AGGTGCTGCAAAGGCAACGAATGC**C**TCTGGTAAAAAAGCTGATGACAAAT	[AG]GG[TC][GA]CT[GA][CT]AAA[GA]G[CAT][TAG][AG][CT][GA]AA[TC]G[CT]**T**TC[TAC]GG[TCA][AC][AG]AAA[AG][GA][CTA][TG]G[AG][TCA]G[AG][CTA]A[AG][AGT]T
crispR5_2	C-A	AACGAGCACGACACCAGAAAGTAA**C**ACAGAAGATAAGTCTCAAAAAGAAG	[AG]A[CTG][GAT]AG[CT][AG]C[GA][AC][ACG]ACC[GAT]GA[AG][AG][GA]TA[AC]**A**[AG]CAGA[ATG]G[AG][CT]AA[GAT]T[CT][TC][CA]AAAAAGA[AT]G
plcR	T-GC	TTATACTTGGACAATCAATACGAA**T**AAGCGCTTTGTCATGCAAATAAAGC	[TC]T[AG]T[AT][TC][TAGC][TC][AG][GA][AG][TC][AG][AG][TGC]C[AG][AG]T[AT][TC][GCA]A[AG]**[GC]**[AG][GA][TG]C[ACTG][CT]T[TA]T[AGT][TC]CA[ATC][GA][TC][AG]AAT[AG][AG][AG]GC
purA	G-A	ATATGTATGTGATACGTCTGTTGT**G**TTAAATGATGCATTAGATAACAATC	[ATG][TC]A[TC][GA]T[AG]TG[TC]GA[TC]AC[GAT]TCTGT[TC]GT**A**[TC]T[AG]AAT[GA]A[TA]G[CT][AG][TC]T[AG]GAT[AG]A[TCA][AG][AG][TAC][CA]
pyc	T-C	AATGCCAGGCGGACAGTACAGTAATTTACAACAACAAGCGAAAG**T**GGTTG	AATGCC[GA]GG[TCG]GG[GATC]CA[GA]TA[TC]AGTAA[TC][CT]T[TACG]CA[GA]CAACA[AG]GC[GATC]AA[GA]G**C**[GACT]GT[TAC]G
rpoB	T-C	GTGGTAGAAGG**T**GATGTTGAGCTGCAATCTATTAAGATTTATGCTCCTGA	GTGGTAG[AC]AGG**C**GA[TA]GTTGAG[CG]TGC[AG]ATC[TGC]AT[TC]AA[GA]AT[TC]T[AG][TC]GC[TG]CC[TACG]GA
SG850	A-C	AGCGGGTGTAGGAA**A**CGGGAAAACAATTGTATATCTTCTATATGCAATTT	[AG]GC[TGA]GG[TGAC]GTAGGAA**C**[ACGT]GG[GTA]AAAAC[ATG]AT[TC]GT[AG]TA[TC]CT[TG][CT]T[AG]TA[TC]GC[AG]ATTT
tpiA	G-TA	TCCAGCTCTATTCTTAGAGCGCCT**G**GTAGCAGCGACTGAAGGAACTGATT	[TG][CA]C[AG]GCT[CT]T[AT]TT[CT][TC]TAGAGCG[CT][CT]T**[TA]**G[TC]AG[CT][AGT][GA][CA][AGTC][AGT]C[TG]GAAGG[AG]AC[TGA]GA[CT]T

*Bold and underlined letters indicate SNP sites. The parentheses indicate that the site is not the only base in *B. cereus* and *B. thuringiensis*

Table S3 Target sequences, RPA primers and crRNAs used for detecting in this study.

**Loci names**	**eProbes**	**Primers**		**crRNA (5’-3’)**
		**names**	**Sequences (5’-3’)**	
tpiA	TCCAGCTCTATTCTTAGAGCGCCT**G***GTAGCAGCGACTGAAGGAACTGATT	tpiA-F	GTTTGCTCTCCAGCTCTATTCTTAGA***TTTC***CT	AAUUUCUACUGUUGUAGAUacCAGGCGCuCuAAGAA
		tpiA-R	CCAAGTACTACGTAGCCTACTTTTAAGTCGCTAAG	
SG850	AGCGGGTGTAGGAA**A***CGGGAAAACAATTGTATATCTTCTATATGCAATTT	SG850reverse-F	GCAAATTGCATATAGAAGATATACAATTGT***TTTC***CCG	AAUUUCUACUGUUGUAGAUccguuuCCuACACCCG
		SG850reverse -R	CTTCCGGAAAAAGGCTTTGAAGAGCGTGATGAAC	
CR5_1	AGGTGCTGCAAAGGCAACGAATGC**C***TCTGGTAAAAAAGCTGATGACAAAT	CR5_1-F	GCAGGTGCTGCAAAGGCAAC***TTTT***GC	AAUUUCUACUGUUGUAGAUgcCuCuGGuAAAAAAGC
		CR5_1-R	CGCTTGTTGATTTCTCTTCTTTTTGAGAC	
CR5_2	AACGAGCACGACACCAGAAAGTAA**C***ACAGAAGATAAGTCTCAAAAAGAAG	CR5_2-F	GAGAAGTCAACGAGCACGACACCAGA***TTTT***AA	AAUUUCUACUGUUGUAGAUaaCACAGAAGAuAAGuC
		CR5_2-R	GTGAAGAAGAACTATTAGATGATTCTGTAGAACC	
Ba813	CCATTGCTAATGTATGCGAATTTC**A***ATTTGCCAAATGACAATTTAGGTTT	Ba813-F	TGTCAGAAACACCATTGCTAATGTATG***TTTA***TTTC	AAUUUCUACUGUUGUAGAUuuucAAuuuGCCAAAuG
		Ba813-R	CTCTAAGAACGGGTATTTCTTTATGCATTTTTG	

Bold and SNP * sites were used to distinguish *B. anthracis* from non-*B. anthracis*. Italic text indicates PAM sequences. Underlined parts indicate the positions of the complementary sequences.

Table S4 RPA reaction conditions (50μL)

Reagent names	Volume (μL)
Primer A(10μmol/L)	2.5
Primer B(10μmol/L)	2.5
2×Reaction Buffer	25
dNTPs(10mM each)	2.5
10×Probe E-mix	5
20×Core Reaction Mix	2.5
DEPC water	6.5
template DNA	1
280mM MgOAc	2.5

Table S5 Cas12a reaction conditions (20μL)

Reagent names	Volume (μL)
DEPC water	12
NEBuffer 3	2
Cas12a	1
Probe	2
crRNA(10μmol/L)	1
PCR/RPA product	2

Table S6 The information table of *Bacillus* species (4947 strains)

Name	Count	Name	Count
Bacillus cereus	1118	*Bacillus flexus*	10
Bacillus thuringiensis	622	*Bacillus fordii*	1
Bacillus mycoides	78	*Bacillus fortis*	13
Bacillus pseudomycoides	111	*Bacillus gaemokensis*	2
Bacillus paranthracis	31	*Bacillus galactosidilyticus*	2
Bacillus paramycoides	3	*Bacillus ginsengihumi*	3
Bacillus cytotoxicus	15	*Bacillus glycinifermentans*	1
Bacillus albus	11	*Bacillus halotolerans*	19
Bacillus bombysepticus	1	*Bacillus haynesii*	3
Bacillus mobilis	9	*Bacillus horikoshii*	1
Bacillus nitratireducens	7	*Bacillus infantis*	5
Bacillus pacificus	8	*Bacillus intestinalis*	7
Bacillus toyonensis	223	*Bacillus korlensis*	1
Bacillus tropicus	12	*Bacillus lentus*	2
Bacillus wiedmannii	166	*Bacillus licheniformis*	186
bacillus sp.	575	*Bacillus luciferensis*	2
Bacillus subtilis	403	*Bacillus mannanilyticus*	1
Bacillus acidiceler	3	*Bacillus marisflavi*	11
Bacillus acidicola	1	*Bacillus massiliosenegalensis*	2
Bacillus acidiproducens	1	*Bacillus megaterium*	139
Bacillus aerius	1	*Bacillus methanolicus*	6
Bacillus aerophilus	2	*Bacillus mojavensis*	3
Bacillus aidingensis	1	*Bacillus nealsonii*	2
Bacillus alkalitelluris	2	*Bacillus panaciterrae*	1
Bacillus altitudinis	61	*Bacillus paralicheniformis*	73
Bacillus amyloliquefaciens	99	*Bacillus pumilus*	156
Bacillus andreraoultii	4	*Bacillus safensis*	50
Bacillus aquimaris	1	*Bacillus shackletonii*	2
Bacillus aryabhattai	23	*Bacillus siamensis*	7
Bacillus atrophaeus	32	*Bacillus simplex*	20
Bacillus aurantiacus	1	*Bacillus smithii*	4
Bacillus azotoformans	2	*Bacillus sonorensis*	8
Bacillus badius	8	*Bacillus swezeyi*	5
Bacillus canaveralius	3	*Bacillus tequilensis*	4
Bacillus cellulosilyticus	1	*Bacillus thermoamylovorans*	6
Bacillus chagannorensis	1	*Bacillus timonensis*	3
Bacillus ciccensis	1	*Bacillus vallismortis*	5
Bacillus circulans	16	*Bacillus velezensis*	130
Bacillus coagulans	39	*Bacillus vietnamensis*	7
Bacillus coahuilensis	3	*Bacillus xiamenensis*	2
Bacillus cohnii	2	*Bacillus zhangzhouensis*	1

Bacillus enclensis	2	*Geobacillus*	44
Bacillus endophyticus	8	*Lysinibacillus sphaericus*	28
Bacillus filamentosus	6	*Bacillus anthracis*	240
*Bacillus anthracis**	12		

*Bacillus anthracis** indicates the suspicious *B. anthracis*.

Table S7 The table of Assembly ID and the rename of *Bacillus cereus* group common strains in this study.

Table S8 The table of Assembly ID and the rename of the representative strains in *B. cereus sensu lato*

Name in this study	Assembly ID
BT01-ATCC10792	GCF_000161615.1
BC01-BC33	GCF_018309165.1
B.wiedmannii-SR52	GCF_008807735.1
B.mycoides-BPN36-3	GCF_018739485.1
B.pseudomycoides-DSM12442	GCF_000161455.1
B.tropicus-AOA-CPS1	GCF_011040455.1
B.cytotoxicus-CH-13	GCF_002250945.2
B.toyonensis-P18	GCF_016605985.1
B.paranthracis-14-9	GCF_010994595.1
B.bombysepticus-Wang	GCF_000831065.1
B.albus-PG26	GCF_004116025.1
B.pacificus-24-6	GCF_010628775.1
B.mobilis-16-00177	GCF_900177005.1
B.paramycoides-LB-RP2	GCF_013391375.1
B.nitratireducens-BM02	GCF_018141025.1
BA01-AmesAncestor	GCF_000008445.1
BA02-Ames	GCF_000007845.1
BA03-Sterne	GCF_000008165.1
BA04-A16	GCF_000512835.2
BC02-ATCC14579	GCF_000007825.1
BC03-ATCC10987	GCF_000008005.1
BC04-AH187	GCF_000021225.1
BC05-CI	GCF_000143605.1
BT02-97-27	GCF_000008505.1
BT03-Al-Hakam	GCF_000015065.1
BT04-HD73	GCF_000338755.1

Genomic DNA concentration

The quantitation of genomic DNA was performed with iQuant™ NGS-BR dsDNA Assay Kit using Qubit 3.0 Fluorometer (Thermo Fisher Scientific Inc., Waltham, MA, USA). DNA concentration were determined following formula (ng/μL $\times 10^{-9}$)/ (5504285×660) = mol/μL = mol/L*10^{6}, 1aM = 10^{-18} mol/L.The results are shown in the Table S9.

Table S9 Genomic DNA concentration

Sample	Concentration (ng/μL)	Concentration (amol/L)
A16R	18.7	5.15×10^{6}
BC307	26.4	7.27×10^{6}
NC7401	22.1	6.08×10^{6}
HD73	15.2	4.18×10^{6}
Bs168	21	5.78×10^{6}

The user manual

Identify_B.anthracis
AGILE V1.1
(Anthracis Genome-based Identification with high-fideLity E-probe)

This version is for Windows users, one-click identification of Bacillus anthracis. Users do not need to write code. Identify_B.anthracis is a tool based python that employs a combination of * in silico * the 9 specific tags of Bacillus anthracis to rapidly identify * B.anthracis * isolates using nucleotide sequencing data.

1. The software can be downloaded from http://github.com/844844/AGILE/ for windows users. Click the code and then Download Zip (Fig. S6).

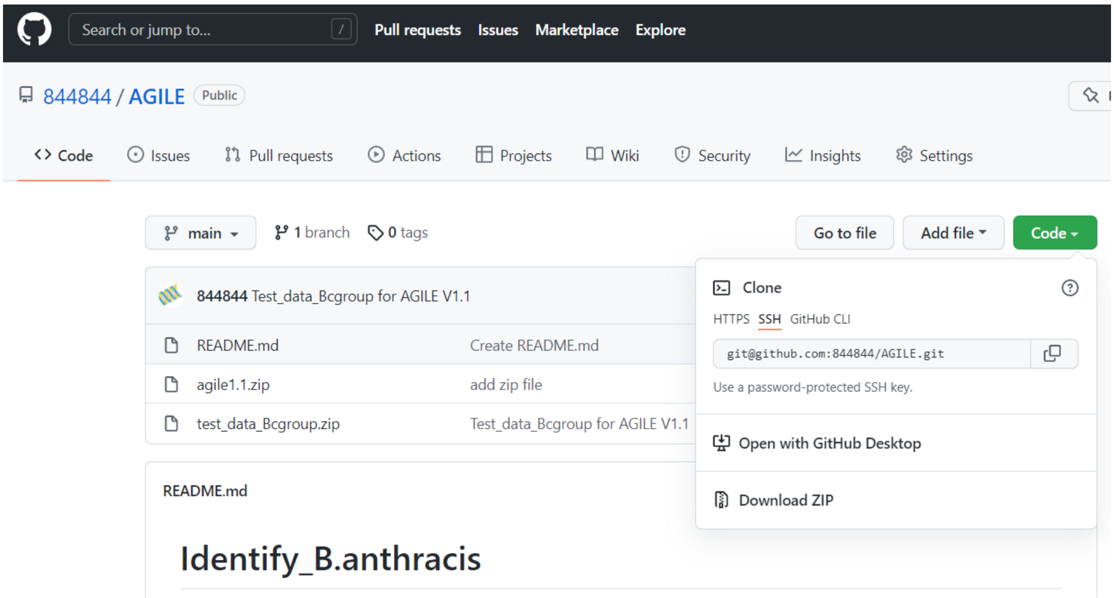

Fig. S6 The website screenshot of Github.

2. Decompress agile.zip, Open agile_start Shortcut (Fig. S7).

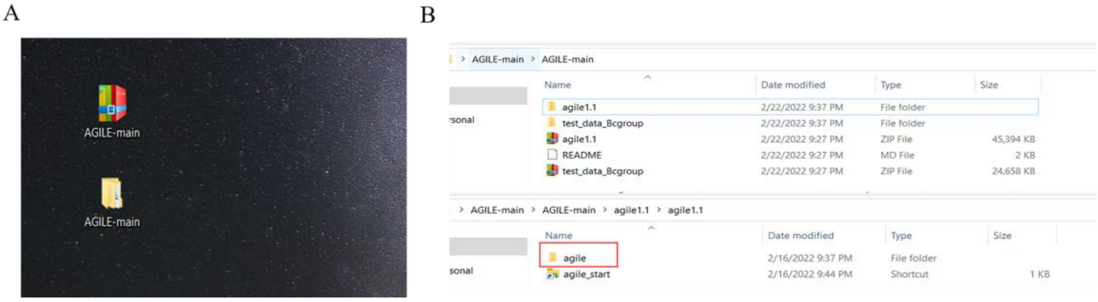

Fig. S7 The website screenshot of decompressing.

3. In Fig. S8, click Select File to open the sequencing file. After entering the sequencing results, clicking open means start identification. Click on the result to export, output the result, and select export location and enter the file name, complete the analysis. The last column in the result file is

the identification result, whether it is Ba or not Ba, deterministic.

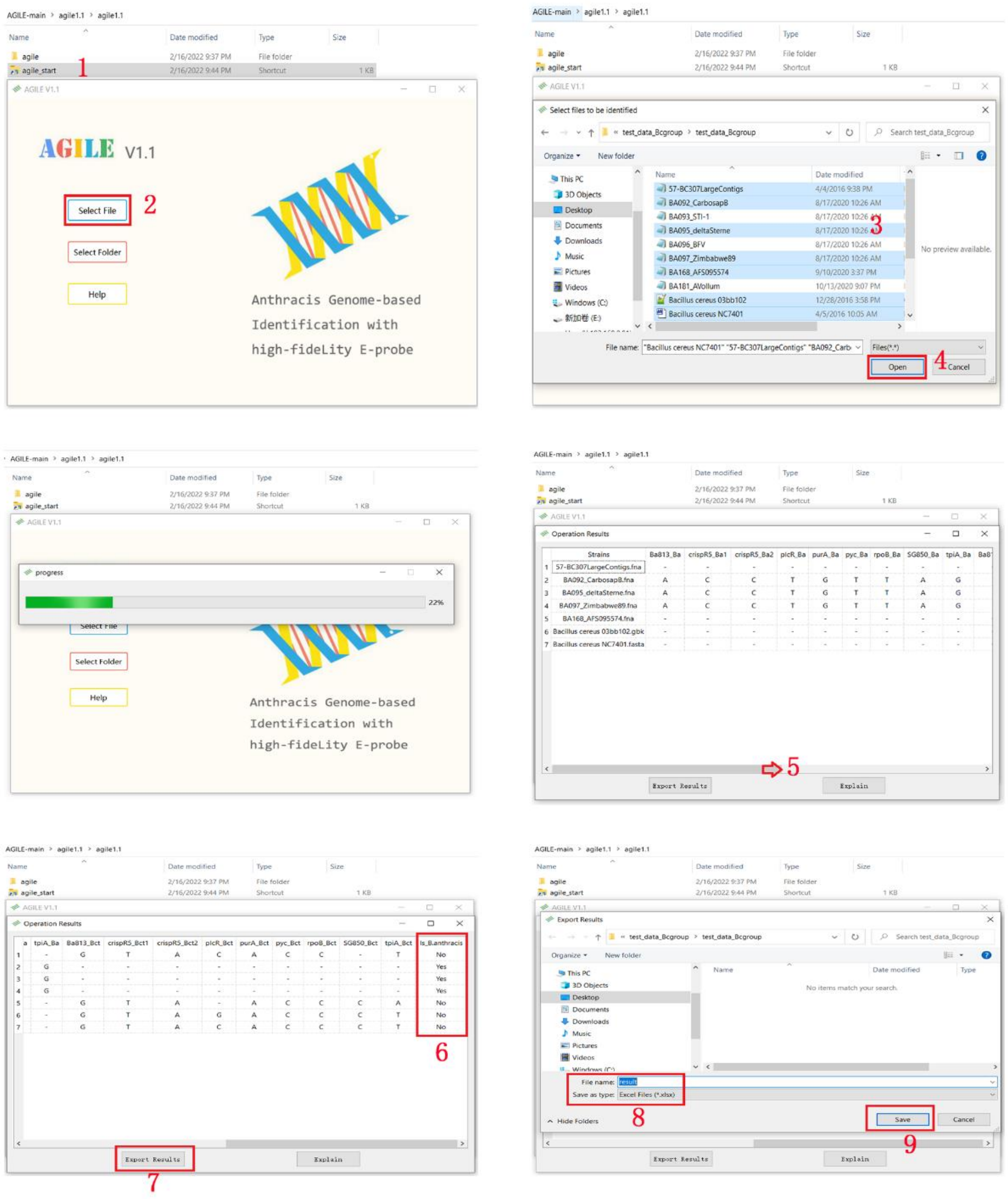

Fig. S8 Workflow of the Select File model.

4. In Fig. S9, click Select Folder to load sequence of whole folder.The common nucleic acid sequences can be loaded. Click on the result to export, output the result, and select export location and enter the file name, complete the analysis. The last column in the result file is the identification result, whether it is Ba or not Ba, deterministic.

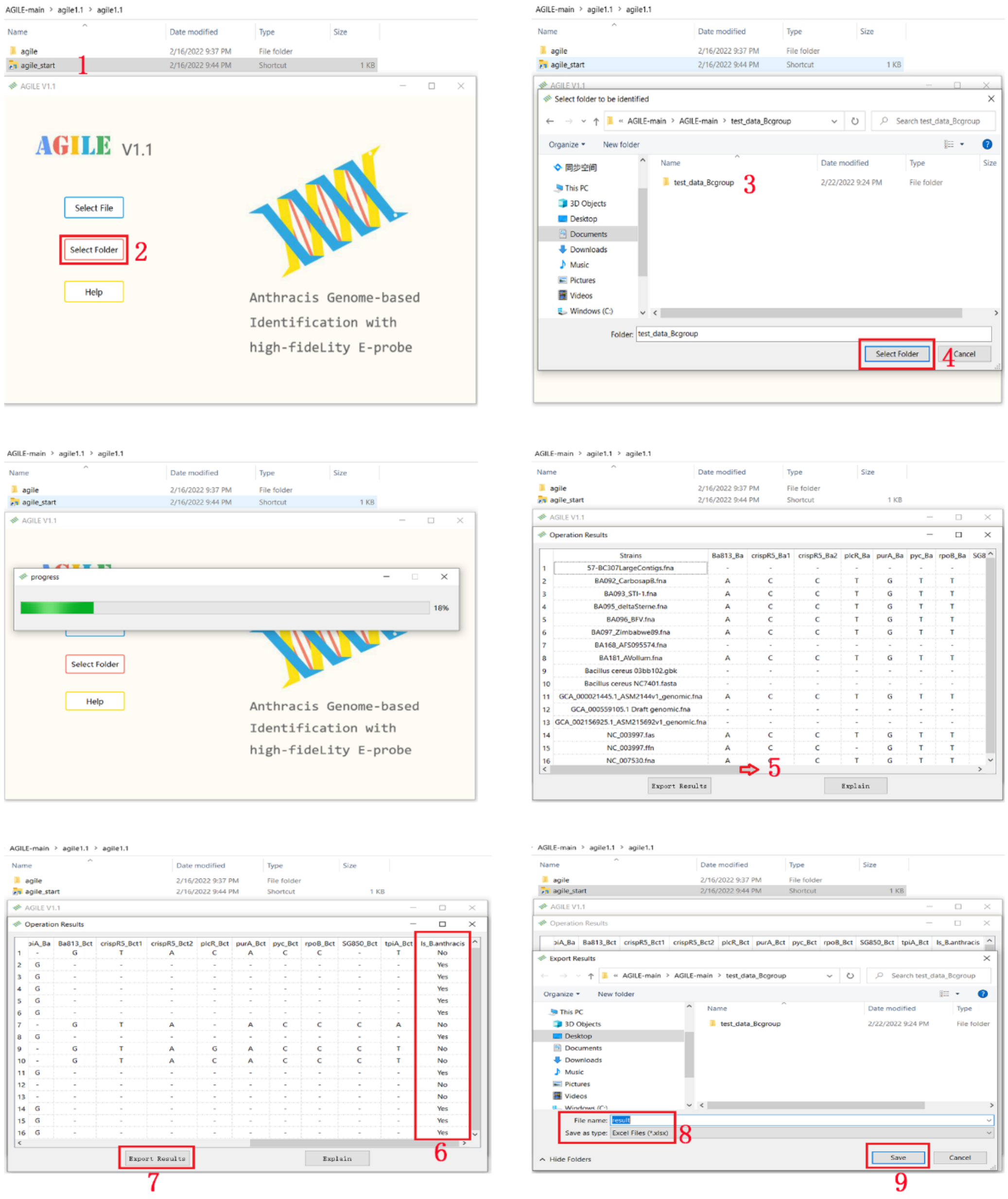

Fig. S9 Workflow of the Select Folder model.

5.Note that office files in .doc, .xls, .ppt and other formats cannot be loaded. The identification result is determined based on whether the specific tags of *Bacillus anthracis* are searched. The more tags are searched, the more reliable the target sample is positive.
